# A *Toxoplasma gondii* Pseudokinase Inhibits Host IRG Resistance Proteins

**DOI:** 10.1371/journal.pbio.1001358

**Published:** 2012-07-10

**Authors:** Martin C. Fleckenstein, Michael L. Reese, Stephanie Könen-Waisman, John C. Boothroyd, Jonathan C. Howard, Tobias Steinfeldt

**Affiliations:** 1 Institute for Genetics, University of Cologne, Cologne, Germany; 2 Department of Microbiology and Immunology, Stanford University School of Medicine, Stanford, California, United States of America; University of Vermont, United States of America

## Abstract

The ability of mice to resist infection with the protozoan parasite, *Toxoplasma gondii*, depends in large part on the function of members of a complex family of atypical large GTPases, the interferon-gamma-inducible immunity-related GTPases (IRG proteins). Nevertheless, some strains of *T. gondii* are highly virulent for mice because, as recently shown, they secrete a polymorphic protein kinase, ROP18, from the rhoptries into the host cell cytosol at the moment of cell invasion. Depending on the allele, ROP18 can act as a virulence factor for *T. gondii* by phosphorylating and thereby inactivating mouse IRG proteins. In this article we show that IRG proteins interact not only with ROP18, but also strongly with the products of another polymorphic locus, *ROP5*, already implicated as a major virulence factor from genetic crosses, but whose function has previously been a complete mystery. ROP5 proteins are members of the same protein family as ROP18 kinases but are pseudokinases by sequence, structure, and function. We show by a combination of genetic and biochemical approaches that ROP5 proteins act as essential co-factors for ROP18 and present evidence that they work by enforcing an inactive GDP-dependent conformation on the IRG target protein. By doing so they prevent GTP-dependent activation and simultaneously expose the target threonines on the switch I loop for phosphorylation by ROP18, resulting in permanent inactivation of the protein. This represents a novel mechanism in which a pseudokinase facilitates the phosphorylation of a target by a partner kinase by preparing the substrate for phosphorylation, rather than by upregulation of the activity of the kinase itself.

## Abstract 2

A secreted kinase from the parasitic protozoan, *Toxoplasma gondii*, is shown to cooperate with a phylogenetically related pseudokinase to phosphorylate and inactivate a mouse resistance protein of the IRG system.

## Author Summary

*Toxoplasma gondii* is an intracellular parasitic protozoan infecting about a third of humankind. Humans, however, are unlikely to be important hosts in terms of the evolution of the parasite because, for completion of the parasite sexual cycle, the infected animal must be eaten by a cat. Therefore, important intermediate hosts for *Toxoplasma* are species like mice that are often preyed on by cats. Mice use an intracellular resistance mechanism, the IRG proteins, against *Toxoplasma*. In turn, *Toxoplasma* appears to have evolved a virulence factor, a protein kinase called ROP18, that inactivates IRG proteins. We show that ROP18 does not act alone. It needs help from the ROP5 pseudokinases, proteins related to ROP18 but without enzymatic activity. ROP5 pseudokinases assist ROP18 by binding to the IRG proteins, holding them inactive, and laying them open to enzymatic attack and likely permanent inactivation by the ROP18 kinase. This mechanism illustrates the principle that members of an enzyme family can lose their enzymatic activity and evolve into regulators or co-factors for the active members.

## Introduction

*Toxoplasma gondii* (*T*. *gondii*) is an intracellular parasitic protozoan of the phylum Apicomplexa, a relative of the malarial Plasmodia. The two groups of human parasites differ greatly in their ecology. In particular, unlike *Plasmodium* species, which have narrow host ranges, *T*. *gondii* is able to establish productive infection in an extraordinary range of intermediate hosts, differentiating to stable encysted forms which can persist lifelong in all warm-blooded animals, birds as well as mammals. In humans, *T. gondii* infection is generally tolerated with minor symptoms, and an estimated 30%–40% of the world's population carry live brain cysts [1]. In mice, however, while *T. gondii* strains of the types II and III clonal lineages are largely avirulent, strains of the type I clonal lineage are highly virulent [2], killing immunologically competent but naïve hosts within 10 d [3].

It has been known for many years that interferon-γ (IFNγ) is essential for resistance against *T. gondii* [4]. A previously underexplored family of IFNγ-inducible large GTPases, the IRG proteins (formerly the p47 GTPases), has been shown to carry much of the responsibility for the successful early resistance of mice to avirulent *T. gondii* strains [5],[6]. Genes encoding four members of the IRG protein family, Irgm1 [7], Irgm3 [8], Irgd [7], and Irga6 [9], have been disrupted and each of the mutant mouse strains shows more or less complete loss of acute resistance to infection with avirulent *T. gondii* strains. Thus the proteins function non-redundantly in resistance, and some mechanistic basis for this non-redundancy has been outlined as follows. The IRG proteins are simultaneously and massively induced by IFNγ [10] in all cell types so far studied and accumulate intracellularly in a GDP-bound inactive state. The inactive state is maintained by three dedicated regulatory members of the family: Irgm1, Irgm2, and Irgm3 [11]. On infection by *T. gondii*, the effector IRG proteins assemble cooperatively, probably in multimers, in a GTP-bound active form over a period of about 60 min on the cytosolic face of the parasitophorous vacuole membrane (PVM) [12],[13]. IRG protein accumulation disrupts the integrity of the PVM [12],[14],[15] and the enclosed parasite is killed for unknown reasons. The IRG resistance system is efficient in protecting mice from types II and III avirulent strains of *T. gondii*, but is defeated by virulent type I strains. IRG proteins largely fail to assemble on the PVM of these strains [13],[16]. Progress has been made recently in understanding the genetic basis for this striking difference of virulence for mice among different genotypes of *T. gondii* and its relationship to the IRG system. Consistent with the complexity of the interaction between a pathogen and its host, forward genetic screens of *T. gondii* have established the existence of several polymorphic loci contributing to virulence, with complex epistatic relations between them [17],[18]. One of these, *ROP18*, encodes a serine/threonine kinase that is secreted into the host cell cytosol at the time of parasite entry. To date, two independent targets of the ROP18 kinase have been identified: the transcription factor ATF6β [19] and the GTPases of the IRG family [20],[21]. ROP18 from virulent type I strains inactivates effector IRG proteins at the PVM by phosphorylation of specific threonine residues in the switch I loop of the nucleotide-binding domain [20],[21].

The present article arises from “pull-down” experiments intended to look for additional *T. gondii* proteins associated with one of the effector IRG proteins, Irga6, in IFNγ-induced cells infected with a virulent type I strain of *T. gondii*. The major components found were ROP5 pseudokinases. *ROP5* was identified as the most statistically significant virulence locus in two independent forward genetic screens, one between type II and type III strains [17], and one between type I and II strains [22]. The *ROP5* locus encodes a cluster of closely related polymorphic genes encoding paralogues of ROP18 kinase [22],[23]. The *ROP5* locus from each strain encodes three different major isoforms, termed ROP5A, ROP5B, and ROP5C. The three isoforms differ markedly between the virulent (type I and type III) and avirulent (type II) loci, and these differences account for 50%–90% of the variation in virulence following intraperitoneal infections of lab strains of mice [22],[23]. Substitution of the critical catalytic aspartate in the kinase active site and the non-canonical structure of the bound ATP suggests that all the ROP5 isoforms are catalytically inactive pseudokinases [24],[25]. Nevertheless, deletion of the *ROP5* cluster from a highly virulent type I strain, RH, yields a remarkably avirulent product [23]. Indeed the RH*Δrop5* strain is even less virulent than the RH*Δrop18* strain, suggesting that ROP5 may be a critical agent of *Toxoplasma*'s manipulation of its hosts [23]. In the present study, we describe the first function for the ROP5 pseudokinases: these proteins act as powerful enhancers of ROP18-dependent phosphorylation of IRG proteins in *T. gondii*-infected cells. This appears to occur in large part by the ability of ROP5 to bind to an interface on IRG proteins required for GTP-dependent activation and assembly on the PVM. Furthermore, when bound to ROP5, the switch I loop of the nucleotide-binding site of the IRG protein is left exposed and susceptible to phosphorylation by ROP18.

## Results

### Irga6 Binds *T. gondii*-Derived ROP5 in Vitro

**Figure 1:**
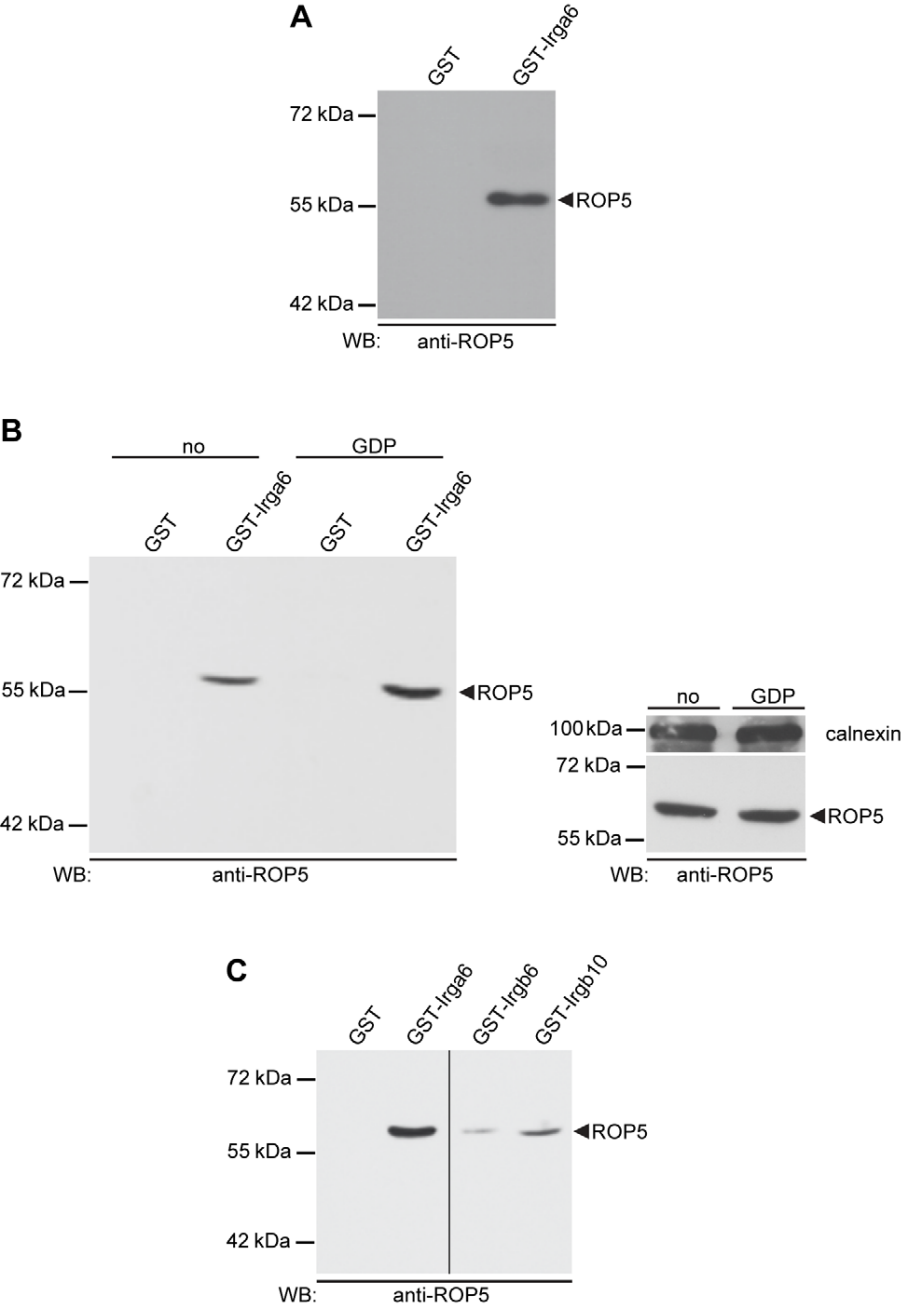
IRG proteins bind *T*. *gondii* virulent strain-derived ROP5 in vitro. Glutathione Sepharose 4B beads loaded at 50 µg protein/100 µl 1∶1 bead suspension with bacterially expressed GST-IRG fusion proteins as bait were incubated at 4°C o/n with whole postnuclear lysates from RH-YFP strain *T. gondii*. Beads loaded with GST served as negative control. Bound proteins were separated by SDS-PAGE and monoclonal antibody 3E2 was used for detection of ROP5 in subsequent Western blot analysis. (A) In vitro pull-down of ROP5 with bacterially expressed and purified GST-Irga6. A single lysate, equivalent to 50×10^6^ organisms per track, was used for GST-Irga6 and GST alone control beads (B) pull-down of ROP5 by Irga6 was markedly enhanced in the presence of 1 mM GDP (left hand blot). Lysates with and without GDP, equivalent to 25×10^6^ organisms per track, were prepared from a single batch of *T. gondii*; the right hand blot shows equal ROP5 signals from the supernatants of the pull-downs with GST alone with or without nucleotide. *T. gondii* calnexin provided the loading controls. (C) GST-Irgb6 and GST-Irgb10 also pulled down ROP5, though more weakly than GST-Irga6. One lysate, equivalent to 50×10^6^ organisms per track, was used. All four tracks were run on a single gel; the vertical line indicates excision of irrelevant tracks.

To identify *T. gondii* proteins interacting with IRG proteins, we prepared Glutathione Sepharose 4B beads loaded with glutathione S-transferase (GST)-Irga6 to pull down proteins from detergent lysates of IFNγ-induced L929 fibroblasts that had been infected 2 h earlier with the virulent *T. gondii* type I strain, RH-YFP. Specificity control was provided by a parallel pull-down with Glutathione Sepharose 4B beads loaded with GST alone. Proteins were eluted from the beads and differential bands from silver-stained gels were analyzed by mass spectrometry (MS). In all differential bands the most abundant *T. gondii* peptides were derived from ROP5. In the band with the highest yield, 60% of peptides originating from *T. gondii* proteins corresponded to ROP5, representing 86% sequence coverage. Peptides unique to each of the three isoforms of ROP5 were identified (Table 1), suggesting that the interaction with Irga6 is a property common to all ROP5 isoforms. ROP5 could also be pulled down from lysates of RH-YFP strain parasites prepared directly from human foreskin fibroblasts as demonstrated both by MS (not shown) and by Western blot (Figure 1A). The efficiency of the pull-down, and therefore presumably the affinity of the interaction between Irga6 and ROP5, was noticeably enhanced when the pull-down was performed in the presence of GDP (Figure 1B, left hand panel). Equal ROP5 content in the lysates with and without GDP was confirmed (Figure 1B, right hand panel). Surprisingly, in view of their substantially different sequences [26], GST fusions of two other IRG proteins, Irgb6 and Irgb10, also pulled down ROP5 from RH-YFP lysates, yielding significant though weaker ROP5 signals in Western blot (Figure 1C). The recovery of all ROP5 isoforms from the Irga6 pull-downs and the interaction of ROP5 with three different IRG proteins suggest that the ROP5 proteins have evolved as a general interactor with the IRG system.

**Table 1:** Peptides of ROP5 isoforms identified by MS after GST-Irga6 pull-down.

ROP5 isoform	Peptides	AA Number
ROP5A	R.G**G**EGILA**R** .H	359–366
	K.RPSL**L**VPGTDSL**S**F**FP**C**A**P**V**PDFV**E**TLI**R** .R	476–504
ROP5B	R.GRGTADGAGVADETHQ**G**PRPPLR.K	104–126
	R.G**D**EGILALHILTAQLIR.L	359–375
	R.LMLGDVSALWK.V	403–413
	R.EFLNASTATFTHALNAWQLGLSIYR.V	431–455
	R.VPGTDSLAFGSCTPLPDFV**K**TLIGR.F	481–505
	R.RLLPLEAMETPEFLQLQNEISSSLSTGQPTAAPSVA.-	514–549
ROP5C (1+2)	R.LLLPSDAVAVQSQPPFAQLSPGQSDYAVANYLLLMPAASVDLELLF**R** .T	302–349
	R. **ST**E**DF**LALHILTAQLIR.L	359–375
	R.LMLGDVS**V**L**R** .K	403–412
	R.EFLNASTATFTHAL**D**AWQLGLSIYR.V	431–455
	R.VPGTDSLAFGSCTPLPDFV**Q**TLIGR.F	481–505
	R.RLLPLEAMETPEFLQLQNEISSSLSTGQPIAAPSVA.-	514–549
ROP5A+ROP5C	R.GDTEAQSGTGDDSDFPQAVAEEVADMSGGR.V	47–76
	R.GRGTADGAGVADETHQEPRPPLRK.R	104–127
ROP5B+ROP5C	R.DLLKREEELIGYCR.E	183–196
	R.EEELIGYCREEALKEPAAMVEAVTATVWPQNAETTVDSLLSQGER.K	188–232
	K.LKLVEPLR.V	234–241
	R.DVERLEDFALK.V	253–263
	R.DVERLEDFALKVFTMGAENSR.S	253–273
	R.TLDFLYVLR.S	350–358
	K.GLVHGHFTPDNLFIMPDGR.L	384–402
ROP5A+ROP5B+ROP5C	R.TNDLASGTPHVAR.G	34–46
	R.VPRVPASSTTTSASEGIFR.R*	77–95
	R.LAQHFR.R	129–134
	R.WLSGLGR.R	147–153
	R.QRPLLDPSFHGLEAGDSFMR.D	163–182
	R.EEALKEPAAMVEAVTATVWPQNAETTVDSLLSQGERK.L	197–233
	R.VGDRSVVFLVR.D	242–252
	R.SVVFLVRDVER.L	246–256
	K.VFTMGAENSR.S	264–273
	R.SELERLHEATFAAAR.L	274–288
	R.LLGESPEEARDRR.R	289–301
	R.LAANLQSK.G	376–383
	R.HILTAQLIR.L	367–375
	R.GPASSVPVTYAPR.E	418–430
	R.VWCLFLPFGLVTPGIK.G	456–471
	R.FLNFDRR.R	506–512

The first three sections (ROP5A, ROP5B, and ROP5C) show peptides that specifically discriminate between the three ROP5 isoforms. Residues in bold type are unique for the specific isoform indicated in the table. Sections 4 (ROP5A+ROP5C) and 5 (ROP5B+ROP5C) show peptides that fit to both indicated isoforms only. The peptides displayed in Section 6 (ROP5A+ROP5B+ROP5C) show peptides that match to all isoforms except that marked with *, which is found in all ROP5 isoforms but ROP5C1. Altogether 51 peptides were found for ROP5C comprising a sequence coverage of 86.2%, 47 peptides for ROP5B comprising a sequence coverage of 70.3%, and 34 peptides for ROP5A comprising a sequence coverage of 55.9%. In total 65 individual peptides for RH-ROP5 were found. Many of those were limited digests of the ones presented in the table and are not shown. The next best hit for any *T. gondii* protein yielded 17 peptides. All together 44 additional *T. gondii* peptides were recovered from this band.

### ROP5 is Required for Efficient Phosphorylation of Irga6 by ROP18

**Figure 2:**
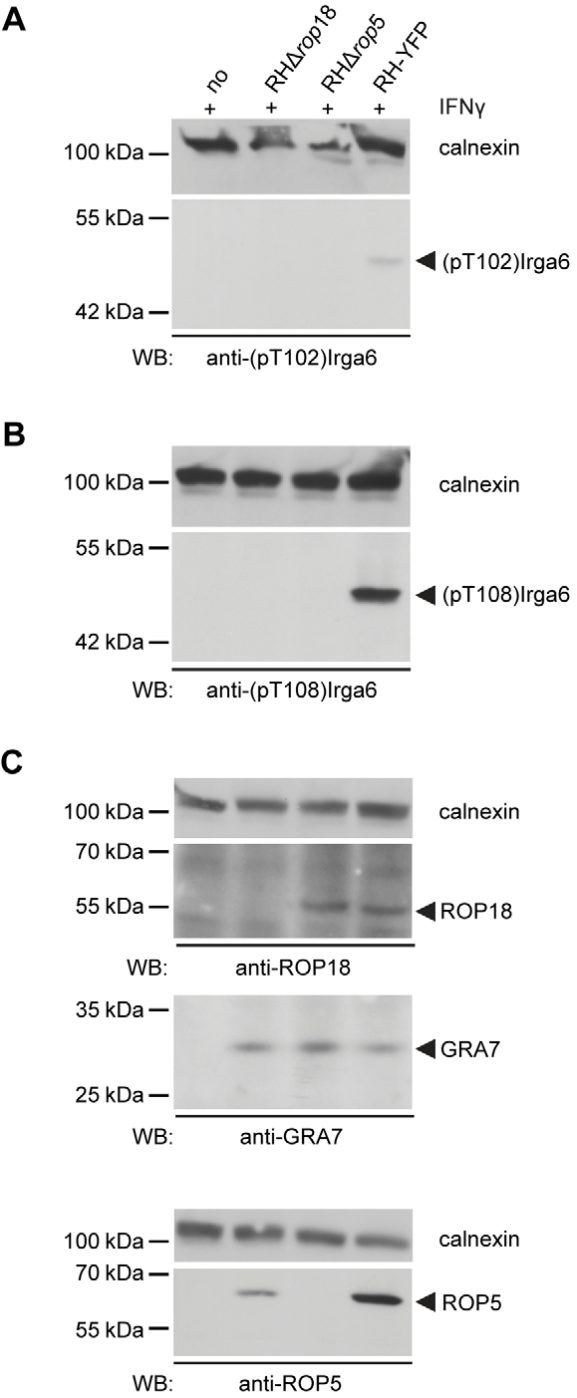
ROP5 is required for efficient ROP18]-[mediated phosphorylation of Irga6. Western blot of detergent lysates from IFNγ–induced L929 cells either uninfected (no) or infected with RHΔ*rop18*, RHΔ*rop5*, or wt RH–YFP *T*. *gondii* strains. Efficient phosphorylation of Irga6 is detectable with (A) anti-phosphothreonine 102 (anti–pT102) and (B) anti–phosphothreonine 108 (anti–pT108) antibodies only after RH–YFP–infection (black arrowheads). (C) Signals for ROP18 (upper panel) and ROP5 (lower panel) are shown in the three infected cell lysates. GRA7 (middle panel) in all infected cell lysates indicates essentially equivalent levels of infection. Calnexin provided loading controls.

ROP5 deficiency renders RH strain parasites completely avirulent in mice [22],[23] and virulence is associated with phosphorylation of IRG proteins by ROP18 [20],[21]. We therefore asked whether ROP5 might be required for Irga6 phosphorylation by ROP18. L929 cells induced with IFNγ were infected with wild-type RH-YFP, RH*Δrop5*, RH*Δrop18*, or left uninfected. Compared with cells infected with wild-type RH-YFP, phosphorylation of the ROP18 target threonines, T102 and T108, was not detectable in RH*Δrop5*- or RH*Δrop18-*infected cells (Figure 2A,B). Expression of ROP18 and ROP5 proteins in cells infected with RH-YFP and the respective deletion strains is shown (Figure 2C). GRA7 content in the three infected cell lysates provided an infection control (Figure 2C). The lack of Irga6 phosphorylation in the RH*Δrop18*-infected cells is consistent with evidence that ROP18 is the main kinase of the RH strain that phosphorylates and inactivates IRG proteins [20],[21], and further suggests that no other virulence kinase in the RH strain can compensate for its loss. The lack of autonomous catalytic activity of ROP5 [25] suggests that this protein acts as a facilitator of phosphorylation of Irga6 by ROP18.

### ROP5 Deficiency Reduces Cell-Autonomous Correlates of Virulence

**Figure 3:**
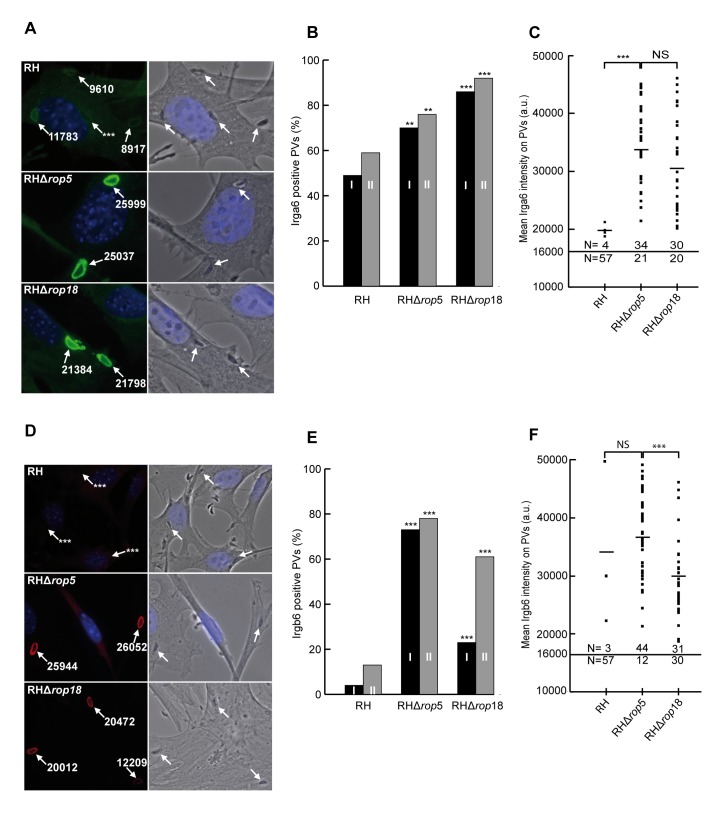
*T. gondii* virulence factors ROP5 and ROP18 inhibit IRG protein binding to the PVM. C57BL/6 mouse embryonic fibroblasts were induced with 3 U ml^−1^ IFNγ for 24 h and infected for 2 h with wt RH strain *T. gondii*, or RHΔ*rop5* or RHΔ*rop18*. Irga6 and Irgb6 positive vacuoles were identified microscopically on slides double stained with mouse mAb 10E7 (anti-Irga6) and 141/1 rabbit antiserum (anti-Irgb6) and appropriate fluorescent tagged species-specific secondary antibodies. (A, D) Left hand panels: fluorescent images of vacuoles from RH, RHΔ*rop5* and RHΔ*rop18* with Irga6 (A, green) and Irgb6 (D, red) fluorescence intensities indicated (white arrows). Right hand panels: phase contrast images. Nuclei stained with DAPI. The fluorescence intensities on vacuoles indicated with *** were too close to the background to be measured. (B, E) The percentage of vacuoles loaded with Irga6 (B) and Irgb6 (E) was determined by visual inspection of coded slides in two separate experiments (I and II, black and grey bars); (C, F) the fluorescence signal intensity of Irga6 (C) and Irgb6 (F) at individual vacuoles was estimated from experiment II (grey bars) of Figure 3B and 3E on coded slides using Volocity automatic software (see Materials and Methods). For technical reasons the automatic threshold for vacuole detection was set at 16,000 pixel units (horizontal line), which loses many vacuoles visibly but weakly loaded with Irga6 (see Figure 3A). Bars across the intensity dot-plots indicate the arithmetic mean intensities of above-threshold vacuoles. *N* values above and below the threshold indicate the number of vacuoles recorded in these two categories. In Figure 3B and 3E, ** and *** indicate numbers of vacuoles loaded with Irga6 and Irgb6, respectively, that are significantly greater than RH at <0.01 and <0.001 levels, respectively (Table S3). In Figure 3C and 3F, *** indicates differences significant at *p*<0.001 in loading intensity of Irga6 and Irgb6 between the indicated data sets by the Mann-Whitney U-test. NS, not significant.

The association of IRG proteins with the parasitophorous vacuole membrane (PVM) is greatly reduced on virulent type I strain vacuoles compared with type II or type III strains [13],[16] and this difference is correlated with lack of phosphorylation of IRG proteins by ROP18 in type II or III strains [20,21]. Since ROP5 is required for phosphorylation of Irga6 T102 and T108 (Figure 2) and has been shown to associate with the PVM ([27] and confirmed in Figure S1) we predicted that loading of IRG proteins onto the vacuoles of RH*Δrop5* parasites would be enhanced relative to the virulent wild-type RH strain. We therefore enumerated by immunofluorescence the loading of vacuoles with Irga6 (Figure 3A,B,C, Table S3) and Irgb6 (Figure 3D,E,F, Table S3) 2 h after infection of mouse embryonic fibroblasts (MEFs) with wild-type RH, RH*Δrop5*, or RH*Δrop18* parasites. For Irga6 (Figure 3B) in two similar experiments (I, II) there were significant increases in the proportions of vacuoles from both RH*Δrop5* and RH*Δrop18* strains that were detectably loaded relative to RH. At the same time, there was also a striking increase in the intensity of loading of Irga6 onto individual vacuoles, with very few RH vacuoles loaded above the technical threshold, and even those few were barely above the threshold (Figure 3C). For Irgb6 (Figure 3E) the proportions of RH*Δrop5* and RH*Δrop18* vacuoles detectably loaded by visual evaluation were dramatically increased relative to RH. However the few residual RH vacuoles loaded carried as much Irgb6 as the RH*Δrop5* vacuoles (Figure 3F). RH*Δrop5* vacuoles were slightly but significantly more heavily loaded with Irgb6 than RH*Δrop18* vacuoles.

Differential effects on loading of Irga6 and Irgb6 onto vacuoles of virulent and avirulent strains have been reported before [13]. For Irga6, the intensity of loading onto virulent type I strain vacuoles is substantially less than on avirulent strain vacuoles, but the proportion of detectably loaded vacuoles is only a little lower. For Irgb6, the proportion of vacuoles detectably loaded is much lower with type I strains but the few remaining loaded vacuoles carry as much Irgb6 as those harboring an avirulent type II or type III strain. Thus the loss of ROP5 converts the IRG loading phenotype of RH vacuoles to that typical of an avirulent type II or type III strain vacuole. The loss of ROP18 had effects qualitatively similar to the loss of ROP5. Thus, ROP5, like ROP18, contributes very significantly to the known phenotypic differences in IRG protein loading onto the vacuoles, which are associated with virulence. The loss of ROP5, however, appears to have a greater impact on Irgb6 loading than the loss of ROP18.

**Figure 4:**
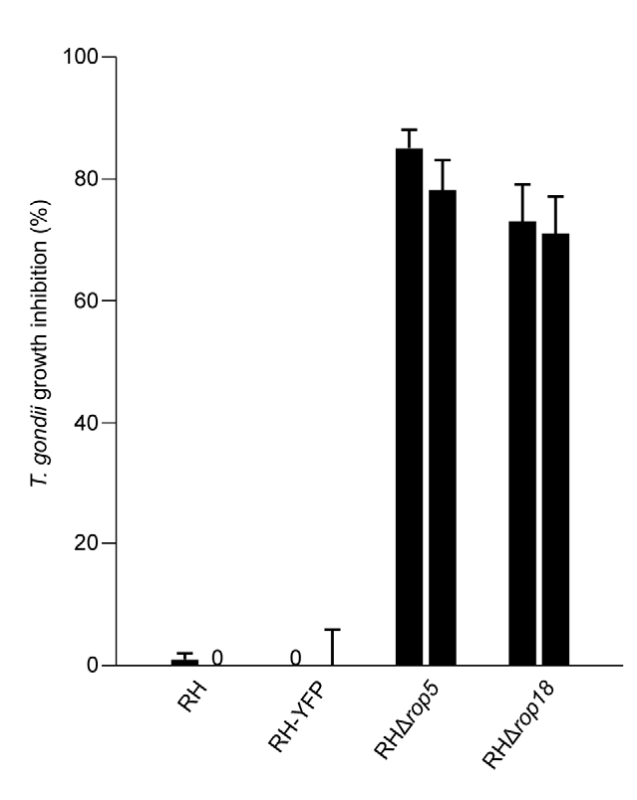
Inhibition of *T. gondii* growth in fibroblasts by IFNγ. C57BL/6 strain mouse embryonic fibroblasts were induced for 24 h with 10 U ml^−1^ IFNγ and infected with RH, RH-YFP, RHΔ*rop5*, or RHΔ*rop18* at an MOI of 0.3. After 24 h, ^3^H-uracil was added and the assay harvested after a further 24 h culture. Growth inhibition was calculated as in Materials and Methods relative to parallel cultures of similarly infected cells not induced with IFNγ. Growth of the RHΔ*rop5* and RHΔ*rop18* parasites was massively inhibited, while RH and RH-YFP were completely insensitive to growth inhibition. Error bars represent the standard deviations of ^3^H-uracil incorporation from triplicate cultures.

We showed earlier that IFNγ treatment of the host cells drastically reduces proliferation of the type II avirulent strain, ME49, compared with the RH strain [28]. This parameter is correlated with IRG protein loading of the PVM and is thus an additional correlate of virulence. We therefore used this assay to assess the in vitro virulence of the RHΔ*rop5* and RHΔ*rop18* strains. The increased loading of IRG proteins onto the PVM in IFNγ-induced cells infected with RH*Δrop5* and RH*Δrop18* (shown in Figure 3) was associated with a striking inhibition of proliferation relative to RH and RH-YFP, measured in vitro by incorporation of ^3^H-uracil (Figure 4). Proliferation of both RH*Δrop5* and RH*Δrop18* parasites was strikingly inhibited by IFNγ compared with RH wild-type and RH-YFP controls.

### Complementation of the RHΔ*rop5* Phenotype in Strains Transgenic for ROP5 Isoforms

**Figure 5:**
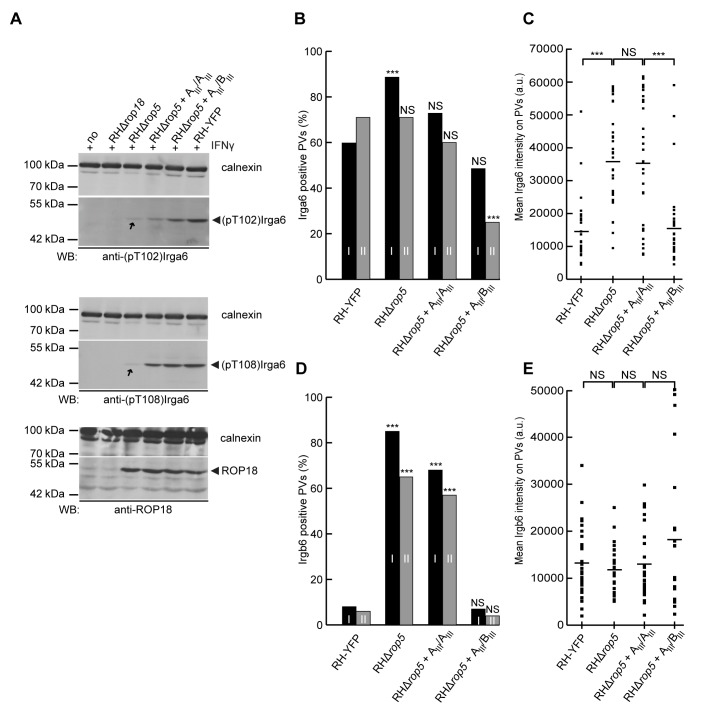
ROP5 isoforms restore virulence-associated functions to RHΔ*rop5*. (A) IFNγ-stimulated L929 cells were left uninfected (no) or infected for 2 h with either of the two deletion strains, RHΔ*rop18*, RHΔ*rop5*, or the RHΔ*rop5* strain transgenic for ROP5 isoforms RHΔ*rop5*+A_III_/A_III_, or RHΔ*rop5*+A_III_/B_III_ (see Materials and Methods for further details) or RH-YFP. Lysates were resolved on SDS-PAGE and stained on Western blots with anti-pT102 (upper panel) and anti-pT108 (middle panel) antibodies. Expression of ROP5 isoform A_III_ in RHΔ*rop5*+A_III_/A_III_ resulted in detectably increased phosphorylation of Irga6 compared with RHΔ*rop5-*infected cells. In cells infected with RHΔ*rop5*+A_III_/B_III_, phosphorylation of Irga6 was restored almost to wt levels. Arrows on the RHΔ*rop5* tracks indicate what is probably residual kinase activity of ROP18 on Irga6 in the absence of ROP5 since these signals are absent from cells infected with RHΔ*rop18* (and unpublished data). Signals of ROP18 serve as infection controls for the ROP18-expressing strains (lower panel). Calnexin serves as a loading control. (B, C, D, E) MEFs were induced with 3 U ml^−1^ IFNγ for 24 h and infected with wt RH, RHΔ*rop5*, RHΔ*rop5*+A_III_/A_III_, or RHΔ*rop5*+A_III_/B_III_ for 2 h in two independent experiments (I and II, black and grey bars) and positive vacuoles were identified with mAb 10E7 (anti-Irga6) (B) or 141/1 rabbit antiserum (anti-Irgb6) (D). Fluorescent signal intensities shown for Irga6 (C) and Irgb6 (E) for individual vacuoles were determined for visually identified positive vacuoles from experiment II as described in Materials and Methods. Presentation of the data is as described in the legend to Figure 3. As already seen in Figure 3, ROP5 deletion and restoration had relatively modest effects on the proportion of vacuoles loaded with Irga6 (5B), while the effects on loading of Irgb6 (5D) were striking. Again as seen in Figure 3, loss of ROP5 caused a highly significant increase in the intensity of Irga6 loading relative to which RHΔ*rop5*+A_III_/A_III_-infection had little effect (5C). However, following infection with RHΔ*rop5*+A_III_/B_III_ the intensity of Irga6 loading was reduced to the levels of wild-type RH. As already seen in Figure 3, loading intensities of the few wild-type RH vacuoles loaded with Irgb6 were the same as for the RHΔ*rop5* vacuoles, as well as for the RHΔ*rop5*+A_III_/A_III_ and RHΔ*rop5*+A_III_/B_III_ vacuoles. Significances shown in Figure 5B and 5D are all relative to the number of Irga6 or Irgb6 loaded vacuoles of the RH strain. Statistical analysis of numerical data for (B) and (D) is given in Table S4. The significances of differences between the datasets shown in Figure 5C and 5E were estimated by the Mann-Whitney U-test.

We next sought to demonstrate that the loss of ROP5 was, indeed, responsible for the strikingly avirulent cell-autonomous phenotype of the RHΔ*rop5* strain. We showed previously that engineered RHΔ*rop5* parasites expressing both of the virulent allelic isoforms, ROP5A_III_ and ROP5B_III_ with epitope tags, were as virulent in vivo as the wild-type RH strain [23]. When L929 cells were infected with these RHΔ*rop5*+A_III_/B_III_ transgenic parasites, Irga6 was phosphorylated on T102 and T108, indicating that these parasites expressed sufficient amounts of the correct ROP5 isoforms to complement the phenotype we had observed for RHΔ*rop5* (Figure 5A). Furthermore, levels of Irga6 and Irgb6 associated with the PVM in MEFs infected with RHΔ*rop5*+A_III_/B_III_ parasites were returned to those characteristic of the wild-type parasites, both in terms of the percentage of vacuoles loaded with Irga6 (Figure 5B) or Irgb6 (Figure 5D) and in terms of the amount of Irga6 (Figure 5C) loaded. For Irgb6, however, and as discussed further above, the amounts of Irgb6 on loaded vacuoles are not increased by deletion of ROP5 and, thus, are not significantly affected upon complementation (Figure 5E). As expected from a previous report [27] ROP5 isoforms A_III_ and B_III_ could be found at the PVM co-localizing with Irga6 in IFNγ-induced MEFs infected with RHΔ*rop5* transgenic parasites (Figure S2).

RHΔ*rop5* parasites expressing two copies of ROP5A_III_ were shown earlier to exhibit a moderate rescue of virulence in vivo [23]. L929 cells infected with these parasites also showed some increase in phosphorylation of Irga6 (Figure 5A) but apparently not enough to inactivate IRG protein activity, because the loading of Irga6 (Figure 5B,C) or Irgb6 (Figure 5D,E) onto the PVM in infected cells was not different from cells infected with RHΔ*rop5* parasites. Very weak phosphorylation of Irga6 is also just visible in the RHΔ*rop5* tracks (Figure 5A) presumably due to residual ROP5-independent activity of ROP18. It is premature, however, to conclude from these results that ROP5B_III_ has a higher potency than ROP5A_III_ in the inactivation of Irga6 and Irgb6, because ROP5A_III_ is expressed at only 10%–20% of the level of ROP5B_III_ in the transgenic parasites [23]. Taken together, these results strongly suggest that the contribution of ROP5 to in vivo virulence is at least in part due to its ability to enhance the phosphorylation of IRG proteins by ROP18.

### ROP5 Binds Irga6 Using a Face Distal from its Pseudoactive Site

To gain insight into the mechanism by which ROP5 acts, we next sought to obtain structural information on the ROP5:IRG complex. To determine the region of ROP5 involved in binding Irga6, we used a nuclear magnetic resonance (NMR) method combining isotopic labeling of specific amino acid types with analysis of chemical shift perturbation upon complex formation. ROP5 and Irga6 are relatively large proteins by NMR standards; while standard NMR methods usually have an upper size-limit of ∼30 kDa, the ROP5 pseudokinase domain:Irga6 complex is ∼90 kDa. We therefore chose to use isotopic labels on specific methyl groups, which have relaxation characteristics superior to the more common amide-labeling scheme.

**Figure 6:**
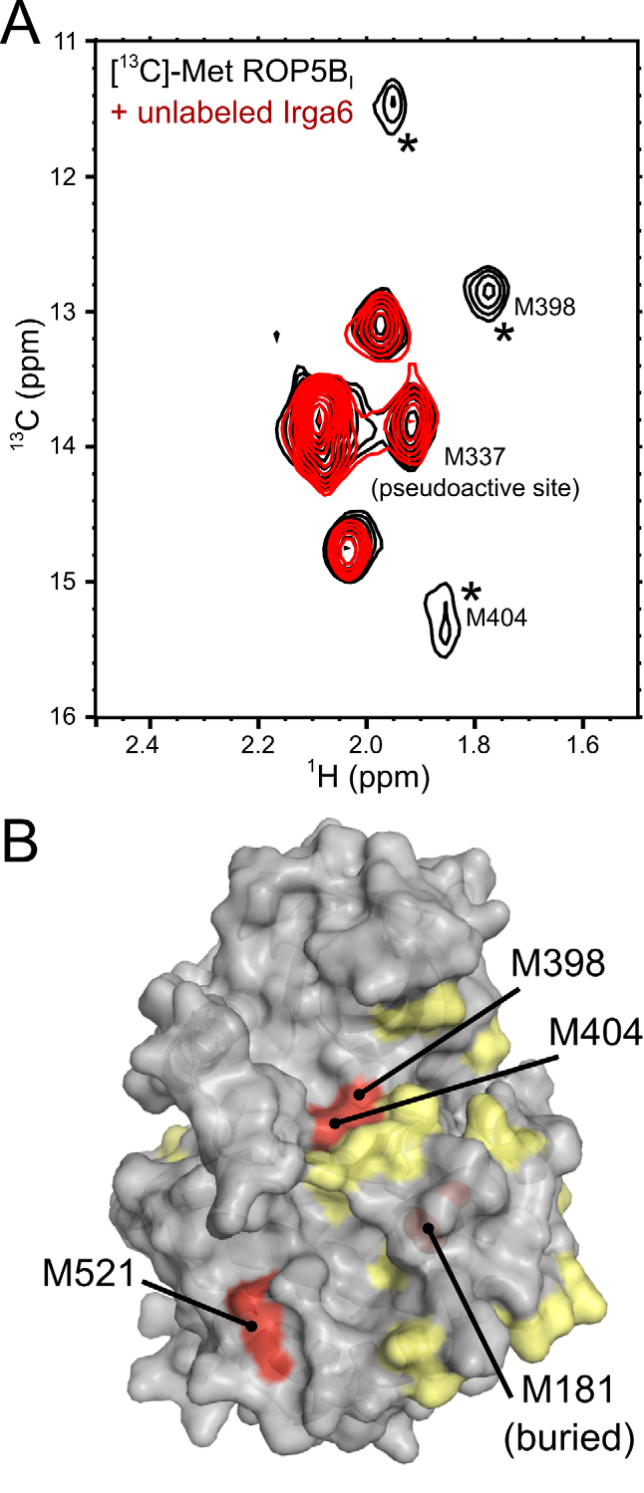
Irga6 binds ROP5 distal from the pseudoactive site. (A) HSQC spectra of [^13^C]-Met labeled ROP5B_I_ in the absence (black) or presence (red) of unlabeled Irga6. Peaks that are broadened upon addition of Irga6 are starred. Peaks that have been assigned by mutagenesis (see Figure S2) are noted. (B) A surface representation of the rear face of the ROP5B_I_ structure, where surface exposed methionines are highlighted in red and the buried methionine M181 is shown in red spheres. Polymorphic residues are highlighted in yellow.

As we have structural information for the pseudokinase domain of ROP5B_I_ (PDB: 3Q60; [23]) and have found that complementation of the RHΔ*rop5* strain with the nearly identical ROP5B_III_ isoform (S535R/S536R are the only substitutions between ROP5B_I_ and ROP5B_III_) enhances the phosphorylation of Irga6 in infected cells (Figure 5A), we began by collecting spectra of ROP5B_I_ that had been specifically [^13^C]-labeled on the C_ε_ of methionines. Spectra of [^13^C]-Met labeled ROP5B_I_ showed well-resolved peaks corresponding to the eight methionines in the sequence (Figure 6A). When unlabeled Irga6 was added, three methionine peaks were broadened, consistent with a complex in slow-intermediate exchange. Such spectral changes could indicate conformational changes near to the residues in question, rather than proximity to a binding interface. However, it is important to note that the ROP5 pseudokinase domain appears to be relatively rigid (which is atypical for a kinase domain); the structures of the ATP-bound and -unbound proteins are virtually identical [25]. Thus the spectral changes observed are most likely due to a direct interaction of residues on the surface of ROP5 with Irga6. The structure of the ROP5B_I_ pseudokinase domain indicates only one surface with a cluster of three methionines that are relatively closely apposed (Figure 6B). To verify this region as involved in the interface, we used mutagenesis to alanine to assign two of the methionines, M398 and M404 (Figure S2B,C). In addition, to verify that the pseudoactive site was not playing a role in the interaction, we also assigned M337 by mutagenesis (Figure S2A). M337 occupies the “gatekeeper” position in the pseudokinase domain, and is thus an ideal probe of the pseudoactive site. As expected, the peak corresponding to M337 is completely insensitive to Irga6 binding (Figure S2A), whereas both M398 and M404 are sensitive. This strongly suggests that Irga6 is binding near M398 and M404 in a surface that includes one other methionine. There are two other methionines near M398 and M404: M181 and M521 (Figure 6B). The side chain of M181 is completely buried, while M521 is highly surface exposed, strongly suggesting that M521 is the third Irga6 sensitive residue. Unfortunately, we were unable to obtain sufficient protein to collect spectra of mutants in either of these two methionines, so the assignment of these latter methionines cannot be proven. Importantly, the region of ROP5 containing M398, M404, and M521 is on the opposite side of the protein from the pseudoactive site. This region of the protein surface is one of the least divergent among the various ROP5 isoforms, consistent with a conserved function and our MS data where peptides specific for all three ROP5 isoforms were recovered from the GST-Irga6 pull-down (Table 1).

### ROP5 Binding Induces Conformational Changes in Irga6

**Figure 7:**
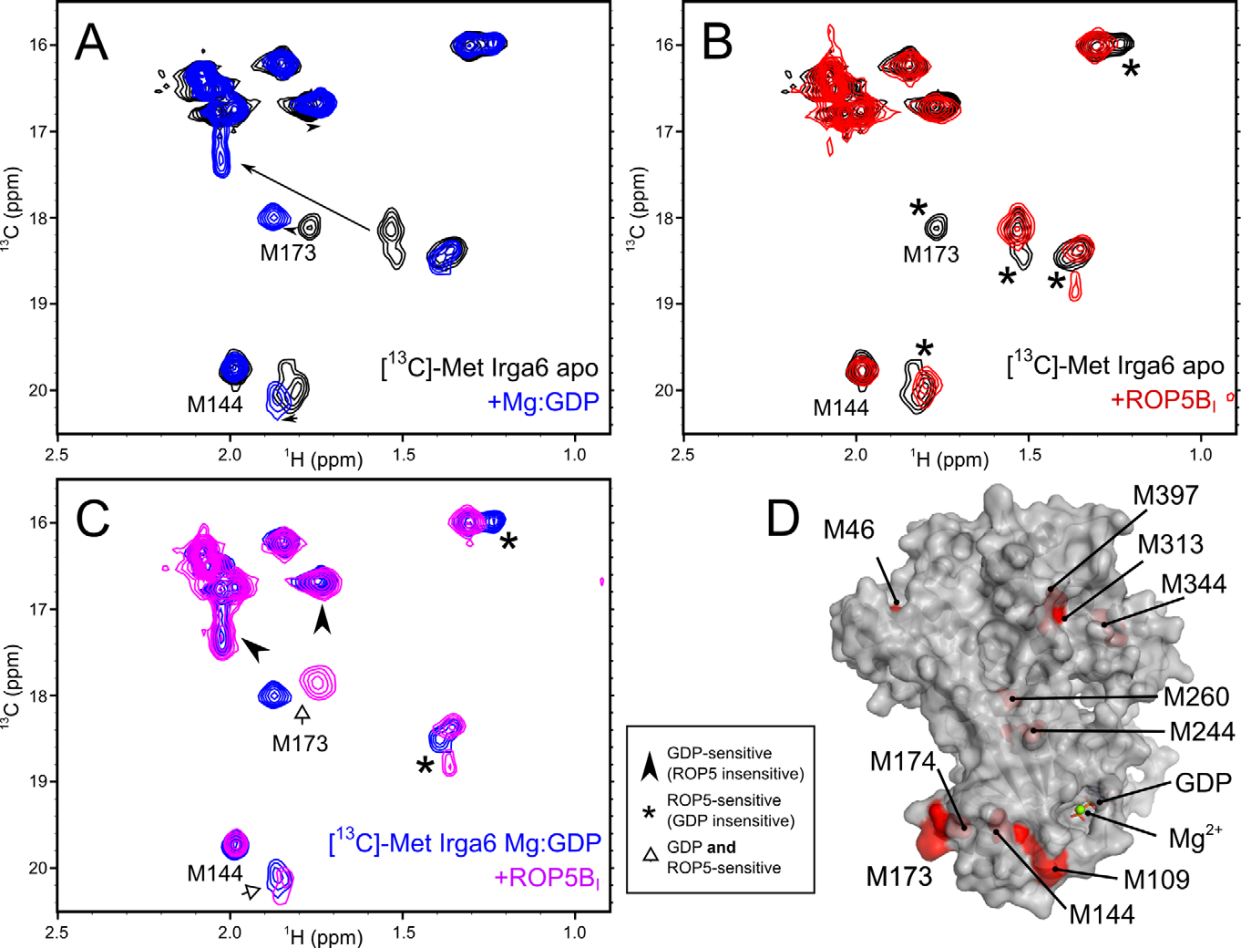
ROP5 binding alters the conformation of the Irga6 active site. (A) HSQC spectra of [^13^C]-Met labeled Irga6 in the absence (black) or presence (blue) of Mg^2+^ and GDP. Peaks that shift are indicated with arrows. Peaks that have been assigned by mutagenesis are indicated. (B) HSQC spectra of [^13^C]-Met labeled Irga6 in the absence (black) or presence (red) of ROP5B_I_, with no Mg^2+^ or GDP. Peaks that exhibit chemical shift changes upon addition of ROP5 are starred. (C) HSQC spectra of [^13^C]-Met labeled Irga6 in the absence (blue) or presence (purple) of ROP5B_I_, with Mg^2+^ and GDP. Peaks that are sensitive to only GDP are indicated with a black arrowhead, those sensitive to ROP5 only are starred, and those sensitive to both are indicated with an open arrow. (D) A surface representation of the GDP-bound Irga6 structure. Surface exposed methionines are indicated in red, and buried methionines are shown as red spheres. The bound Mg^2+^ is shown as a green sphere and the bound GDP as sticks.

To identify the region of Irga6 that interacts with ROP5, we collected spectra using [^13^C]-Met labeled Irga6. Four methionines (M109, M144, M173, and M174) surround the nucleotide-binding site in Irga6 (PDB: 1TPZ; [29]). We first sought to identify peaks whose chemical shifts were sensitive to the presence of nucleotide. We collected spectra of [^13^C]-Met Irga6 in the presence and absence of GDP and Mg^2+^. Three peaks showed substantial changes to chemical shift upon addition of nucleotide, and a fourth peak showed minor changes (Figure 7A). The spectra of nucleotide-free ^13^C-Met Irga6 show several peaks with shoulders, suggesting multiple conformations in slow exchange. Two of these peaks are also sensitive to the presence of nucleotide, consistent with crystallographic data indicating partial stabilization of the Irga6 apoprotein structure in presence of GDP [29].

We next collected spectra of [^13^C]-Met labeled Irga6 in the presence of ROP5B_I_ but without nucleotide. As seen in the above experiments with labeled ROP5, several peaks exhibited substantial chemical shift perturbations (Figure 7B). The fact that we observe peaks that are broadened as well as those that are shifted suggests that two processes, with slightly different rates, are occurring. Thus, in the ROP5:Irga6 complex, Irga6 appears to undergo some conformational change, in contrast to the conformational stability of ROP5. Interestingly, each of the peaks exhibiting a shoulder in the apo-spectra is sensitive to interaction with ROP5, suggesting that ROP5 may restrict the global conformational dynamics of Irga6. Among the peaks that exhibited the most substantial changes upon ROP5 binding were two of the nucleotide-sensitive peaks. As we did not observe a substantial number of other spectral changes, it is also likely that ROP5 binds near this site. Consistent with this interpretation, when both ROP5 and GDP are present, two peaks exhibit chemical shifts that are distinct from those seen for the apoprotein, or for the individual ligand-bound states (i.e., only ROP5 or GDP). We assigned the peaks corresponding to M173 and M144 by mutagenesis to alanine (Figure S2D,E). While M144 (which is buried) is largely insensitive to both ROP5 and nucleotide, the peak corresponding to the surface-exposed M173 undergoes the largest additional chemical shift changes upon ROP5 binding (Figure 7). The second labile peak is not yet assigned.

Taken together, these data suggest that ROP5 binds close to the activation interface on the nucleotide-binding domain and tends to stabilize a conformation related to the GDP-bound conformation. This would suggest that ROP5 affinity for Irga6 may be sensitive to the nucleotide-bound state of the IRG protein, and indeed as noted above, we observed an increase in ROP5 recovered from an Irga6 pull-down assay when GDP was present (Figure 1B), consistent with a model for the interaction in which the binding of ROP5 to Irga6 stabilizes (and is stabilized by) the GDP-bound, inactive conformation of the protein.

### Identification of the ROP5-Binding Interface on Irga6 by Mutagenesis

**Figure 8:**
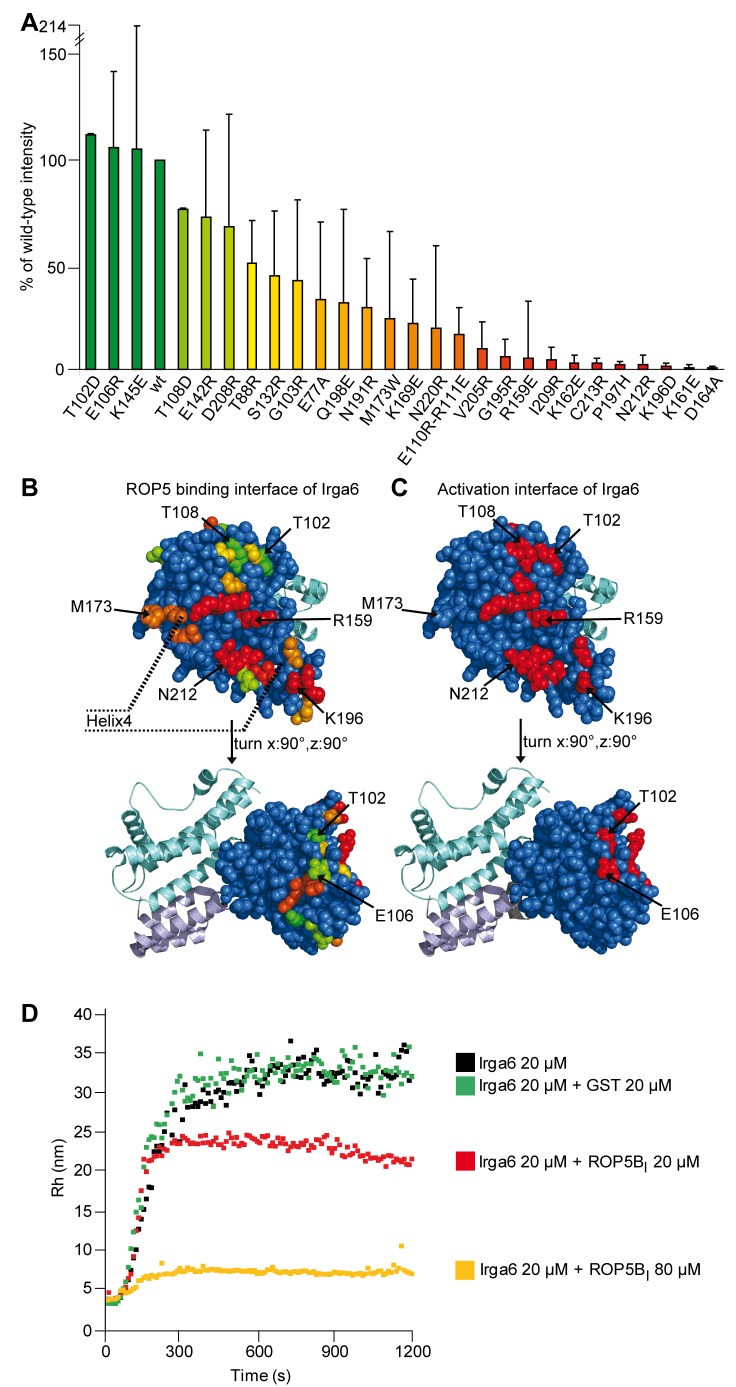
The activation interface of Irga6 largely contributes to interaction with ROP5. (A) In vitro pull-down of ROP5 with bacterially expressed and purified wt or mutant GST-Irga6 (at 25 µg protein/100 µl 1∶1 bead suspension) from RH-YFP strain *T. gondii*-lysates. GST was used as a negative control. Values reflect the mean ROP5 signal intensity and standard deviations of 2–3 pull-down experiments based on quantitated Western blots using 3E2 antibody (anti-ROP5). The signal given by GST was subtracted and the value obtained for each mutant normalized against wt Irga6. The values were correlated with a color in a spectrum ranging from green (100% of wt intensity) to red (0% of wt intensity). (B) Monomeric structural model of Irga6 based on Irga6-M173A in the GDP-bound form (PDB 1TQ6, [29]). The G-domain with the residues analyzed in (A) is depicted as spheres colored according to the values given in Figure 8A. Residues not assayed for inhibition of ROP5 binding are shown in blue. The rest of the protein is shown in ribbon structure (N-terminal helical domain in light blue, C-terminal helical domain in cyan). (C) Irga6-M173A-GDP in the same orientations as Figure 8B, with the residues contributing to the activation interface [30] shown in red. (D) ROP5B_I_ inhibits oligomerization of Irga6 in vitro. GTP-dependent oligomerization of 20 µM wt Irga6 was monitored by dynamic light scattering (DLS) in the presence or absence of equal or 4-fold molar excess amounts of ROP5B_I_ pseudokinase domain protein. Values shown give the mean hydrodynamic radius of molecular complexes in solution. Monomeric Irga6 in the presence of GDP has a hydrodynamic radius of 3.4 nm. Oligomerization of wt Irga6 is almost completely inhibited by addition of a 4-fold molar excess of ROP5B_I_.

Irga6 is activated by binding of GTP, followed by oligomerization [30], and its association with the PVM is prevented by mutations that interfere with GTP-dependent activation of the protein [31]. GTP-dependent oligomerization depends on the formation of a symmetrical dimer interface between two Irga6 monomers involving the nucleotide-binding site in the G-domain, followed by reciprocal trans-hydrolysis of the GTP to GDP [31]. The NMR data suggested that ROP5 also binds at or near the nucleotide-binding site. We therefore used pull-down assays from RH-YFP detergent lysates to determine the binding of ROP5 to GST-Irga6 proteins mutated at essential, surface-exposed residues on the activation interface; ROP5 binding to the mutant Irga6 proteins was then assayed from scanned Western blots. Mutation of five residues (K161, K162, D164, K196, and P197) on the activation interface blocked ROP5 binding >95% (Figure 8A,B,C). By additional mutagenesis we further defined an extension of the activation interface on Helix 4 not included in previous studies [29],[31]. Helix 4 residues are required for oligomerization (unpublished data), and mutation of three of these residues (E209, N212, and C213) also blocked ROP5 binding >95% (Figure 8A,B,C). Mutations of residues elsewhere on the activation interface had no or only a weak impact on ROP5 binding, the latter perhaps due to conformational effects (Figure 8A,B,C). Thus, the binding site of ROP5 overlaps with, but is clearly distinct from, the Irga6 activation interface (compare Figure 8B,C). Particularly striking is that mutations of the two threonines, T102 and T108, that are targets for ROP18 phosphorylation on the conformationally active switch I loop did not affect ROP5 binding, suggesting that ROP5 binding would be compatible with the kinase activity of ROP18 on Irga6 occurring in a trimeric complex.

### ROP5 Directly Inhibits GTP-Dependent Oligomerization of Irga6

The surface on Irga6 to which ROP5 appears to bind overlaps with the Irga6 dimer interface required for GTP-dependent oligomerization and activation. We therefore asked whether purified ROP5 protein would act as a direct competitive inhibitor of Irga6 oligomerization/activation. Irga6 GTP-dependent oligomerization was assayed by dynamic light scattering. While GTP-dependent oligomerization of Irga6 was unaffected by the presence of GST as an inert protein control, addition of the ROP5B_I_ pseudokinase domain inhibited Irga6 oligomerization in a concentration-dependent manner, with a modest effect at 20 µM (equimolar with Irga6) and essentially complete at 80 µM (Figure 8D). Similar results were obtained with ROP5B_III_ (unpublished data).

### ROP5 Isoforms Enhance ROP18-Mediated Phosphorylation of Irga6 in Vitro

**Figure 9:**
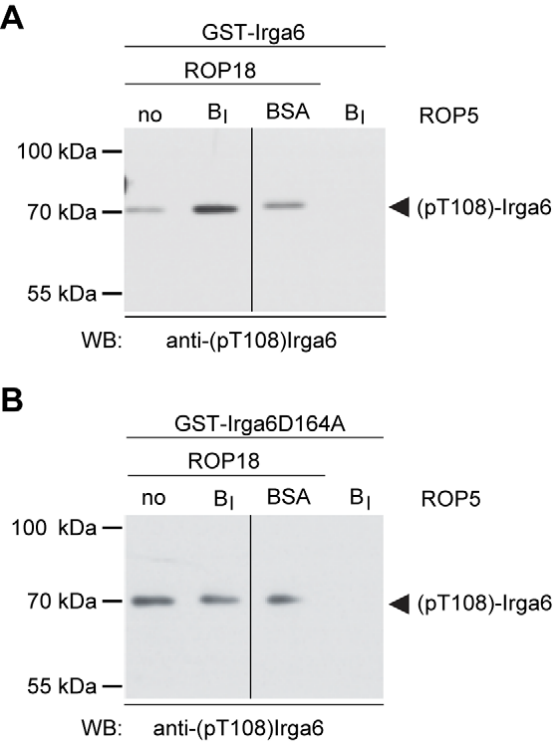
Binding of Irga6 to ROP5 is required for enhanced ROP18-mediated phosphorylation of Irga6 in vitro. Bacterially expressed and purified wt GST-Irga6 (A) or the GST-Irga6-D164A mutant (B) that is unable to bind ROP5 (see Figure 8A) and GST-ROP18-Ty were incubated in the absence or presence of purified pseudokinase domain protein ROP5B_I_ in an in vitro kinase reaction supplemented with 1 mM ATP (left panels). Each kinase reaction was analyzed by Western blot for phosphorylation of Irga6 using anti-pT108 antibody. (A) Enhanced ROP18-mediated phosphorylation of wt Irga6 can be detected after addition of ROP5B_I_. (B) With Irga6-D164A mutant the presence of ROP5B_I_ does not result in enhanced phosphorylation. Addition of BSA instead of ROP5B_I_ served as a control for an effect of total protein in the reaction system (right panels). In the absence of ROP18 there was no detectable phosphorylation of either wt Irga6 or Irga6D164A (right panels). Vertical lines indicate excision of irrelevant tracks from the images of the two gels.

We showed earlier that purified, recombinantly expressed full-length, doubly-tagged GST-ROP18-Ty can phosphorylate purified Irga6 in vitro [21]. In view of the suggestion from the mutagenesis results that the ROP5 interaction surface does not overlap the threonine targets for phosphorylation by ROP18 (Figure 8A,B,C), we asked whether the conformation of ROP5-bound Irga6 is a better substrate for ROP18 in vitro than the unbound conformer. We therefore added purified ROP5B_I_ pseudokinase domain protein to in vitro ROP18-Irga6 kinase reactions. As shown in Figure 9A (left panel), Irga6 phosphorylation on T108 was modestly enhanced by addition of equimolar amounts of ROP5B_I_. To determine whether this increase was due to an effect of ROP5 on ROP18, or an interaction between ROP5 and Irga6, we repeated the experiment using an Irga6 mutant protein, D164A, that does not interact with ROP5 (see Figure 8A). No increase in phosphorylation on T108 of the mutant protein was seen (Figure 9B, left panel) compared with the irrelevant protein, BSA (right panels), implying that the enhanced phosphorylation of Irga6 by ROP18 depends on the physical interaction of ROP5 with Irga6. The enhancement of ROP18 phosphorylation by ROP5 in this in vitro system with purified protein reagents is clear (Figure 9A), but far smaller than that seen in cells infected with virulent parasites (compare Figure 9A and Figure 5A). Technical issues may contribute to this quantitative discrepancy. For example, the in vitro experiments were conducted in solution with recombinantly expressed protein that lacks N-terminal Irga6 myristoylation [32],[33], and the interaction between Irga6 and both ROP5 and ROP18 probably occurs at the PVM when the IRG protein tends to activate and oligomerize.

An interaction between ROP18 and Irga6 was shown recently in a co-precipitation protocol by Fentress et al. [20] and a direct interaction by in vitro kinase activity with purified proteins [21]. We can supplement these results from a pull-down experiment using GST-Irga6 as bait with RH-YFP lysate (Figure S3). This latter result is further confirmed by mass-spectrometric analysis of similar pull-down material at the predicted molecular weight of ROP18, yielding 19 ROP18 peptides with a coverage of 36% (unpublished data). The participation of other *T. gondii-*derived proteins, including ROP5, in these interactions is under investigation.

## Discussion

The polymorphic, secreted ROP5 rhoptry pseudokinases are known to play an important part in virulence of *Toxoplasma gondii* for mice [17],[22],[23], but their mode of action was previously unknown. We have shown in this study that the protein products of virulent alleles of ROP5 are able to bind to at least three effector members of the IRG family of mouse resistance GTPases, Irga6, Irgb6, and Irgb10, and we have identified the probable interaction surfaces on ROP5 and on Irga6. The interaction of Irga6 with ROP5 in infected cells greatly enhances ROP18-mediated phosphorylation of two critical threonines on the conformationally active switch I loop, close to the nucleotide-binding site, a modification shown in earlier studies to be strongly associated with virulence of type I strains [20],[34]. We show that ROP5 plays an essential role in determining the parameters of cell-autonomous virulence in vitro that correlate with in vivo virulence. The parasitophorous vacuolar membranes (PVM) of the RHΔ*rop5* strain, lacking ROP5 but carrying a competent allele of the virulent ROP18 kinase, are almost as heavily loaded with IRG proteins in IFNγ-induced mouse cells as are typical avirulent strains; this phenotype is completely complemented in RHΔ*rop5* strain transgenic for virulent alleles A_III_ and B_III_ of ROP5. Furthermore, the intracellular replication of RHΔ*rop5* strain parasites is as efficiently controlled by IFNγ treatment of the cells as is the replication of avirulent strains. Taken together, earlier results, showing that virulent alleles of ROP18 are required for efficient IRG phosphorylation [20],[21], and the present results, showing that phosphorylation by ROP18 is conditional on the presence of virulent strain ROP5, offer a simple explanation for the previously enigmatic result that type II strains encode an almost certainly functional ROP18 but IRG accumulation at the PVM proceeds unabated: they carry alleles of *ROP5* that are ineffective at assisting ROP18 in the phosphorylation of IRG proteins. Similarly, the fact that type III strains are mouse-avirulent despite the fact that they carry a high-virulence allele of *ROP5* is probably largely due to the virtual null allele of *ROP18* in these strains: expression levels of *ROP18* transcript are several orders of magnitude lower in type III strains than in types I or II, and introduction of a type I or II allele of *ROP18* into a type III strain increases virulence by more than a thousand-fold [17],[18].

The NMR results suggest an IRG-binding surface on the ROP5 protein that is not conformationally affected by the interaction with IRG proteins. This surface is distant from the pseudokinase active site, and is largely conserved between ROP5 isoforms, consistent with the evidence that all three isoforms of ROP5 were recovered from pull-downs from type I lysates with Irga6 (Table 1). The putative ROP5-binding surface on Irga6 partially overlaps the recently defined activation interface on Irga6 whose integrity is required for GTP-dependent oligomerization and the activation of hydrolysis [31]. This surface is conformationally modified by GTP-binding to create a stable dimerization interface that is only resolved by GTP hydrolysis. The NMR data suggest that the binding of ROP5 to Irga6 may favor the inactive GDP-dependent conformation of Irga6, an interpretation supported by the increased efficiency of ROP5 interaction with Irga6 in the presence of GDP. Whether ROP5 imposes further conformational modifications on Irga6, as suggested by the NMR results, or only traps the GDP-bound conformation, is not yet clear. Since the putative interaction surface of ROP5 on Irga6 physically overlaps with the activation interface, it was no surprise that purified ROP5 pseudokinase domain could directly block GTP-dependent oligomerization in vitro. Thus competition for the shared interaction surface between Irga6 and ROP5 could by itself contribute to inactivation of the IRG protein. However, it is striking that T102 and T108, the two essential, conformationally labile, conserved switch I loop targets for phosphorylation by ROP18 kinase, are apparently not involved in the interaction with ROP5. It therefore seems likely that upregulation in IRG-specific ROP18 kinase activity by ROP5 is caused, at least in part, by the same mechanism by which ROP5 inhibits Irga6 multimerization; that is, that ROP5 binds adjacent to the nucleotide-binding site and stabilizes a conformation of Irga6 in which the two target threonines, T102 and T108, on the switch I loop can be preferentially phosphorylated. Structural data on the ternary complex is needed to determine whether ROP18 directly contacts ROP5 in the complex.

**Figure 10:**
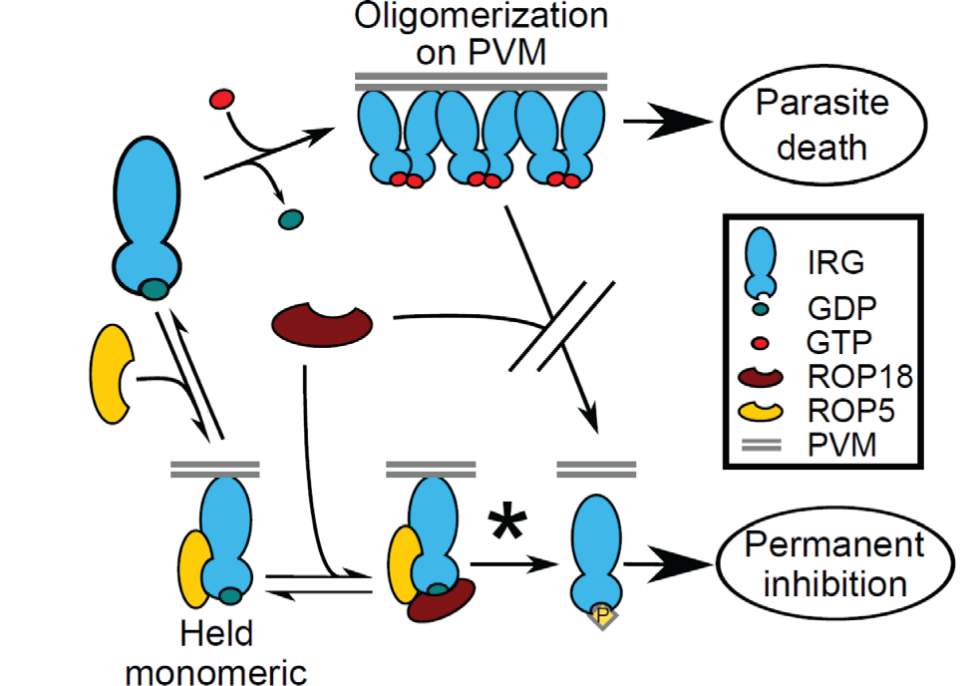
A model for ROP-mediated inhibition of IRG function. ROP5 slows the rate of IRG oligomerization, holding it in a GDP-bound monomeric conformation that facilitates phosphorylation by ROP18. According to this model, ROP18 is unable to phosphorylate the GTP-bound activated IRG protein and thus unable to prevent oligomerization at the PVM. It is important to note that all steps are in equilibrium except for phosphorylation (starred). It is also important to note that both ROP5 and ROP18 associate with the PVM through their RAH domains, which, for clarity, we have not included in the figure.

A plausible scenario (Figure 10) for the interaction of Irga6, ROP5, and ROP18 should be played out on the PVM, where Irga6, ROP5, and ROP18 are largely confined in the infected cell. From the known affinities of Irga6 for GTP (15 µM) and GDP (1 µM) [30] and the cytosolic concentrations of the two nucleotides of ∼300 µM and 100 µM, respectively [35], Irga6 is predicted to be predominantly in the monomeric, GDP-bound state in the cytosol at equilibrium. Immediately after entry of the parasite, IRG proteins begin to arrive at the PVM, probably by simple diffusion while still in the GDP-bound state, bind transiently to the membrane via an unknown mechanism, and activate locally by GTP-dependent interaction with neighboring IRG molecules. Irga6 is myristoylated and the myristoyl group is essential for stable interaction with the PVM. Furthermore, there are indications that the myristoyl group is conformationally interactive with the nucleotide-binding site and is probably exposed by GTP-binding [33] as is the case with Arf GTPases [36]. Although IRG binding to the vacuole is detectable as early as 2.5 min after parasite entry [13], it is likely that ROP5 will initially be in excess relative to the IRG proteins, allowing it to bind and slow their activation. The IRG:ROP5 complex is in equilibrium with the unbound states, and as the IRG protein concentration at the PVM rises, the molar ratios become less favorable for ROP5 and free IRG proteins would tend to activate and oligomerize. However efficient phosphorylation of T102 and T108 proteins by ROP18 in ROP5-assisted complexes will cause permanent inactivation. Without activation of Irga6, the myristoyl group would not interact with the membrane and the inactivated protein would diffuse back into the cytosol, resulting in reduced loading of Irga6, as observed at the virulent vacuole (Figure 3 and [21]).

Although purified ROP18 can phosphorylate IRG proteins in solution ([21] and this article, Figure 9A,B) it is difficult to assess how relevant the in vitro conditions are under which this ROP5-independent kinase activity is displayed. Certainly, as we show in this article, ROP18 is scarcely able to phosphorylate IRG proteins in infected cells in the absence of sufficient ROP5, while the presence of virulent ROP5 without ROP18 is not sufficient to confer virulence. Thus, ROP5 and ROP18 appear to act in concert to inactivate the IRG system, ROP5 perhaps holding IRG in an inhibited form just long enough for ROP18 to make the fatal phosphorylation. The complex in which ROP5 and ROP18 both interact with IRG proteins is not clear. From the proximity of the ROP5 binding site on Irga6 to the switch I loop, ROP18 could interact directly with ROP5 during phosphorylation. In an alternative scenario the conformational change in Irga6 caused by ROP5 binding would facilitate access of the ROP18 kinase to its target threonines, without requiring a direct interaction between the two ROP proteins. Further structural and kinetic information is required to resolve this.

Recently, ROP18 has been shown also to phosphorylate the ER-stress regulator, ATF6β, in infected cells, a target unrelated to the IRG family with a putative effect on virulence [19]. Since our data demonstrate that ROP5 acts directly on the IRG family, rather than globally upregulating the kinase activity of ROP18, it is unlikely to play a role in other ROP18 functions. It is likely, however, that ROP5, like ROP18, also targets other host factors. Indeed, genetic data conclusively demonstrated that ROP5 has both ROP18-dependent and -independent effects on virulence [17]. This conclusion can also be drawn from the fact that the deletion of *ROP5* attenuates virulence of type I strains much more than the deletion of *ROP18*, which would not be predicted if the only role of ROP5 was to potentiate the action of ROP18 [23]. Thus it will be interesting to determine what partners, if any, ROP18 requires for its phosphorylation of ATF6β and ROP5 requires for its other, still unknown functions. Note that the functional heterogeneity of ROP5 within a given strain need not imply a variety of different molecular targets since it could as well be explained by the necessity to accommodate to the considerable structural diversity of IRG proteins as by the targeting of completely unrelated substrate molecules.

It is clear that the IRG resistance system of mice looms remarkably large in the evolution of *T. gondii* virulence. Virulence differentials associated with ROP5 and ROP18 polymorphism together account for about 90% of the presently known virulence differentials for mice between the three clonal lineages. It is therefore likely that the mouse is an evolutionarily important host for *T. gondii* and that there have been a number of reciprocating steps in the co-evolution of *T. gondii* virulence for mice, via the ROP kinases and pseudokinases, and mouse resistance against *T. gondii*, via the IRG resistance mechanism. The polymorphism of the ROP5 and ROP18 virulence systems is likely to be confronted by polymorphic resistance systems in intermediate hosts that find themselves under selective pressure from the parasite. Our own recent work confirms the existence of a widespread polymorphism in the IRG proteins of the mouse associated with differential resistance to *T. gondii* strains carrying different virulence alleles (unpublished data). *T. gondii* evolution will have accelerated in recent millenia due to the exceptional abundance of the domestic cat as a definitive host. The traditional prey animals of the domestic cat are likely to have borne the brunt of this ecological shock. The surprising finding that both ROP5 and ROP18, the two principal polymorphic *T. gondii* virulence factors known for the mouse, target the IRG resistance system provides strong evidence that small rodents such as the mouse or other IRG-expressing animals are important intermediate hosts in the complex world-wide ecology of *T. gondii*.

## Materials and Methods

### In Vitro Passage of *T. gondii* Strains and Infection of Mouse Fibroblasts

Tachyzoites from type I virulent strains RH [37], RH-YFP [38], and the following deletion and transgenic strains (described in [23]), RHΔr*op5* (in which the *ROP5* locus has been deleted) and RHΔ*rop18* (in which the *ROP18* locus has been deleted) and the transgenic strains RHΔ*rop5*+A_III_/A_III_ (carrying two differentially tagged copies of the ROP5A isoform from type III strain CTG), RHΔ*rop*5+A_III_/B_III_ (carrying differentially tagged ROP5A and B isoforms from type III strain CTG) were passaged in confluent monolayers of HS27 human foreskin fibroblasts. Immediately after harvest, tachyzoites were used for infection of mouse fibroblasts at various multiplicities of infection or lysed for subsequent pull-down experiments.

### Cell Culture

L929 (C3H/An strain) mouse fibroblasts and embryonic fibroblasts prepared from 14 d C57BL/6 embryos (MEFs) were cultured in DMEM, high glucose (Invitrogen) containing 2 mM L-glutamine, 1 mM sodium pyruvate, 1× MEM non-essential amino acids, 100 U ml^−1^ penicillin and 100 µg ml^−1^ streptomycin supplemented with 10% FCS (Biochrom). Unless indicated otherwise, cells were stimulated with 200 U ml^−1^ IFNγ (Cell Concepts) for 24 h before infection.

### In Vitro Kinase Assay

Bacterially expressed and purified wt GST-Irga6 protein [30] or the activation interface mutant D164A GST-Irga6 [31] were incubated for 15 min at 30°C in 25 mM Tris/HCl (pH 7.5), 15 mM MgCl_2_, and 1 mM ATP with purified GST-ROP18-Ty protein [21] in the absence or presence of the purified pseudokinase domain of ROP5B_I_ [25]. The kinase reaction was stopped by boiling the samples in sample buffer (80 mM Tris/HCl (pH 6.8), 5 mM EDTA, 4% SDS, 34% sucrose, 40 mM DTT, and 0.002% bromophenol blue) for 5 min at 95°C. Proteins were resolved by SDS-PAGE and analyzed by Western blot.

### Tachyzoite and Mouse Cell Post Nuclear Lysate Preparation

L929 fibroblasts, derived from C3H/An mice, were used for these experiments in preference to C57BL/6 MEFs because of their reproducible behavior and superior growth characteristics. L929 fibroblasts (4×10^5^) were seeded in 2 ml DMEM in individual wells of a six-well plate and stimulated with 200 U ml^−1^ IFNγ for 24 h. Two hours after infection cells were harvested by scraping in PBS, collected by high-speed centrifugation, and lysed in 100 µl NP-40-lysis buffer (0.5% NP-40, 150 mM NaCl, 20 mM Tris/HCl (pH 7.6), 2 mM MgCl_2_, 2 mM DTT supplemented with protease and phosphatase inhibitors (Roche)) for 30 min on ice. Postnuclear lysates were resolved by SDS-PAGE and analyzed by Western blot.

*T*. *gondii* RH-YFP strain parasites (25 to 100×10^6^) were lysed in 500 to 800 µl NP-40-lysis buffer (0.1% NP-40) in the presence or absence of 2 mM GDP for 2 h at 4°C with gentle agitation. Postnuclear lysates were used in pull-down experiments.

### Immunoreagents

Immunoreagents used in this study were: 3E2 mouse monoclonal antibody against ROP5 isoforms [39], anti-ROP18 rat antiserum (El Hajj and Dubremetz, unpublished), affinity-purified rabbit sera 87558 against (pT108)Irga6 and 87555 against (pT102)Irga6 [21], 165/3 rabbit antiserum [40], and 10E7 mouse monoclonal antibody against Irga6 [33], 141/1 rabbit antiserum against Irgb6 [13], 201.1 rat antiserum against *T. gondii* GRA7 (unpublished), and rabbit anti-calnexin antiserum (Calbiochem). Alexa 448 and Alexa 555 labeled donkey anti-mouse and anti-rabbit fluorescent antisera (Molecular Probes), goat anti-mouse-HRP (Pierce), donkey anti-rabbit-HRP (GE Healthcare), and goat anti-rat-HRP (Jackson Immuno Research Laboratories) polyclonal antibodies were used as secondary antibody reagents.

### Pull-Down Assays and Gel Preparation for Mass Spectrometric Analysis

An in vitro pull-down approach was used to find interactions between Irga6 and *Toxoplasma gondii* (*T. gondii)*-derived proteins. 25 or 50 µg (see figure legends) of purified GST, GST-Irga6, GST-Irgb6 or GST-Irgb10 was mixed with 100 µl 1∶1 bead suspension of Glutathione Sepharose 4B (GE Healthcare) resin in 500 µl PBS containing 2 mM DTT for 1 h under continuous rotation. The resin was collected by high-speed centrifugation, washed 3× with ice-cold lysis buffer containing 2 mM DTT without detergent, and incubated with 500 to 800 µl of lysate from *T. gondii-*infected, IFNγ-induced L929 cells or freshly harvested *T. gondii* organisms o/n at 4°C under continuous rotation. In some experiments GDP was added to 1 mM in all buffers. Beads were washed 3× with ice-cold lysis buffer without detergent and boiled for 5 min at 95°C in SDS-PAGE sample buffer. Released proteins were resolved by 10% SDS-PAGE. Gels were either silver-stained or analyzed by Western blot. For band intensity measurements after Western blot, ImageQuant TL (version 7.0, GE Healthcare) was used. Protein models were created using PyMOL (version 0.99, DeLano Scientific).

For identification of Irga6 binding partners, polyacrylamide gels were silver-stained. Gels were fixed with 40% ethanol/10% acetic acid for 30–60 min. After washing in H_2_O, the gels were sensitized with 0.2% sodium thiosulfate in 30% ethanol/6.8% sodium acetate for 30 min followed by several washes with H_2_O and incubated in 0.5% silver nitrate for 30 min. After several washes with H_2_O, gels were developed with 0.0185% formaldehyde in 2.5% sodium carbonate/0.0024% sodium thiosulfate and the reaction was terminated with 0.5% glycin. All steps were performed under gentle agitation. Bands of interest were immediately cut out and analyzed by mass spectrometry or stored at 4°C.

### Mass Spectrometric Analysis

#### Tryptic in-gel digest

After dehydration of minced bands in 100% acetonitrile (ACN), proteins were reduced 2× with 10 mM DTT in 10 mM NH_4_HCO_3_ for 45 min at 56°C and alkylated with 55 mM iodacetamide in 10 mM NH_4_HCO_3_ at RT in the dark. After dehydration in 100% ACN, gel pieces were equilibrated with 10 mM NH_4_HCO_3_ containing porcine trypsin (12.5 ng ml^−1^; Promega) on ice for 2 h. Excess trypsin solution was removed and hydrolysis was performed for 4 h at 37°C in 10 mM NH_4_HCO_3_. Digests were acidified with 5% trifluoroacetic acid (TFA), and peptides were extracted with 0.1% TFA followed by extraction with 60% ACN/40% H_2_0/0.1% TFA followed by a two-step treatment using 100% ACN. Extractions were combined, concentrated by vacuum centrifugation, and desalted according to [41].

#### Nano-LC ESI-MS/MS

Experiments were performed with an LTQ Orbitrap Discovery mass spectrometer (Thermo) coupled to a Proxeon Easy-nLC II. Intact peptides were detected in the Orbitrap at 30.000 resolution in the mass-to-charge (m/z) range 350–2,000 using m/z 445.120025 as a lock mass. Up to 10 CID spectra were acquired following each full scan. Peptides were separated on a 10 cm, 75 mm C18 reversed phase column (Proxeon) over 74 min at a flow rate of 250 nl min^−1^ (buffer A: 0.1% formic acid, 2% ACN in H_2_O; buffer B: 0.1% formic acid in ACN).

#### Peptide and protein identification

Mascot 2.2 (Matrix Science) was used for protein identification by searching the complete proteome database of *T. gondii*, *Mus musculus* or *Escherichia coli* (provided by the European Bioinformatics Institute, 06/2011). Mass tolerance for intact peptide masses was 10 ppm for Orbitrap data. Mass tolerance for fragment ions detected in the linear ion trap was 0.8 Da. Carbamidomethylation of cysteines was set as a fixed modification, and oxidation of methionines was set as a variable modification.

### Oligomerization Assay

Oligomerization of purified Irga6 (20 µM) was measured by dynamic light scattering (DLS, DynaPro molecular sizing instrument, Protein Solutions) in presence of 10 mM GTP as described previously [31].

### Expression, Mutagenesis and Purification of Recombinant Proteins

Expression constructs were generated and site-directed mutagenesis performed as described earlier [31]. Many of the Irga6 mutations used in this article were from this previous study [31]. Mutations made for the present study are listed with the mutagenic primer sequences in Table S1. Recombinant proteins GST-Irga6, GST-Irgb6, GST-Irgb10 and GST alone were expressed from pGEX-4T-2 constructs in *Escherichia coli* BL21 strain following o/n induction with 0.1 mM IPTG at 18°C. Cells were lysed in PBS/2 mM DTT containing protease inhibitor (Complete Mini EDTA free, Roche) using a microfluidiser (EmulsiFlex-C5, Avestin). Lysates were cleared by centrifugation at 50,000 g for 60 min at 4°C and loaded on a GSTrap FF Glutathione Sepharose affinity column (GE Healthcare) in PBS/2 mM DTT. Proteins were eluted with 10 mM reduced L-glutathione in PBS/2 mM DTT and the protein containing fractions subjected to size exclusion chromatography (Superdex 75, Superdex 200; GE Healthcare).

For purification of Irga6 the N-terminal GST tag was cleaved off by o/n incubation of the resin with thrombin (Serva) at 4°C. After elution, Irga6 was further purified by size exclusion chromatography (Superdex 75, GE Healthcare). In each case fractions containing the target protein were concentrated using a Vivaspin 20 centrifugal concentrator (Sartorius).

An abbreviated protocol of purification was used for experiments involving multiple mutant Irga6 proteins. After protein expression the cleared lysate was incubated with 2 ml of Glutathione Sepharose 4B beads (GE Healthcare) in a fritted column (Bio-Rad) o/n at 4°C. After washing 3× with 10 ml PBS/2 mM DTT, the protein was eluted with 10 mM reduced L-glutathione without prior cleavage of the N-terminal GST tag. The eluted fusion proteins were concentrated using a Vivaspin 20 centrifugal concentrator (Sartorius) and washed 3× with PBS/2 mM DTT to minimize the amount of L-glutathione. A comparison of the native and experimental N-termini of the IRG proteins used in this study is given in Table S2. Expression and purification of GST-ROP18-Ty was performed as described recently [21].

### NMR Methods

All spectra were collected at 30°C on a Varian Inova 800 MHz spectrometer equipped with a conventional probe. Binding was monitored using ^13^C-^1^H Heteronuclear single quantum correlation (HSQC) experiments with a spectral width of 30 p.p.m. in the ^13^C-dimension and 10 p.p.m. in the ^1^H-dimension. 512 complex points were collected in the direct dimension and 128 complex points were collected in the indirect dimension with between 4 and 16 scans per increment, depending on protein concentration. Data were transformed in VNMRJ 3.0 software (Agilent) and analyzed in Sparky (UCSF).

### Immunocytochemistry

Fixation and staining of C57BL/6 mouse embryonic fibroblasts (MEFs) grown on coverslips was performed as described earlier [32]. MEFs were used in preference to L929 cells for these experiments because of their superior characteristics for optical microsocopy. Intracellular parasites were identified from the pattern of staining of the *T. gondii* protein GRA7 [42],[43]. Quantification of IRG protein signal intensity at the *T. gondii* PVM was either determined manually from digital photographic images as described and justified in detail previously [13] or automatically on digital photographic images using the Volocity 3D Image Analysis Software (version 5, Perkin Elmer) (see individual figure legends). Microscopy and image analysis was performed essentially according to [13]. All microscopic assessments, whether manual or automated, were performed blind on coded slides.

The significances of differences in the frequencies of IRG-loaded vacuoles (Figures 3B,E, 5B,D) were analyzed statistically by pairwise comparisons using Fisher's exact test to accommodate the small data sets (Tables S3 and S4). Differences between the intensities of loading of individual vacuoles under different conditions were analyzed by the Mann-Whitney U-test for ranked data.

### *T. gondii* Proliferation Assay

*T. gondii* growth in infected C57BL/6 mouse embryonic fibroblasts was determined using the ^3^H-uracil incorporation assay [44] at several multiplicities of infection (MOI) as described previously [45]. ^3^H-uracil was added 24 h after infection and cells were harvested 24 h later.

## Supporting Information

Figure S1 Co-localization of Irga6 and ROP5 at the PVM. MEFs were induced with 100 U ml^−1^ IFNγ for 24 h and infected with either RHΔ*rop5* complemented with (A) ROP5 A_III_-HA+ROP5 A_III_-FLAG or (B) with ROP5 A_III_-HA+ROP5 B_III_-FLAG for 2 h prior to fixation. The cells were stained with rabbit anti-Irga6 serum (165) and mouse anti-FLAG (M2) monoclonal antibody. Images were taken in the green channel for Irga6 (100 ms) and in the red channel for ROP5-FLAG (200 ms). Since the expression of the ROP5A isoform is considerably weaker than ROP5B [22], we also added a technically enhanced image of the red channel in (A). Since the RHΔ*rop5* strain complemented with the A_III_ and B_III_ isoforms strongly reduces Irga6 loading, we added an enhanced image for the Irga6 channel in (B). The enhanced images were used for the merge. White arrows indicate areas of strong co-localization.
(EPS)

Figure S2 Assignment of ROP5 and Irga6 spectra by mutagenesis. (A) Spectra of [^13^C]-Met-labeled wild-type ROP5 (black) and the mutant M337A (purple) are overlayed. One peak is missing in the mutant spectra, which thus corresponds to M337, and has been circled. For the mutants M398A (B, red) and M404A (C, blue), in addition to the missing peak, other peaks have shifted. As M398 and M404 pack against each other, this is not unexpected. Taken together, the most likely assignments are circled, and shifts marked with arrows. (D) Spectra of [^13^C]-Met-labeled wild-type Irga6 (black) and the mutant M173A (red) are overlayed. In addition to the loss of several “shoulder” peaks (which are not GDP-sensitive), one major peak (which is both ROP5- and GDP- sensitive, see Figure 7) disappears, which has been circled. (E) Spectra of GDP-bound [^13^C]-Met-labeled wild-type Irga6 (black) and the mutant M144A (red) are overlayed. As M144 is buried, one might expect slight chemical shift perturbations to peaks corresponding to nearby residues. Indeed, in addition to the one missing peak (circled), likely corresponding to M144, we also observe small chemical shift perturbations in other nucleotide-sensitive peaks (arrows). Of note, M173 appears to be sampling two distinct states in slow exchange, as it is resolved by two distinct peaks.
(EPS)

Figure S3 ROP18 is pulled down as an Irga6 interaction partner from RH-YFP lysate. Lysates from 30×10^6^ RH-YFP or RH*Δrop18* parasites were incubated with GST-Irga6 or GST control beads. Bound proteins were eluted and prepared for Western blot with anti-ROP18 antiserum. (A) The 55 kDa band of ROP18 pulled down by GST-Irga6 but not by GST alone from RH-YFP but not from RH*Δrop18* is indicated. The heavy band marked * is a reproducible but unexplained cross-reaction of the rat anti-ROP18 antiserum with GST-Irga6. (B) RH-YFP and RH*Δrop18* lysates used in (A) were assayed directly in Western blot for the expression of ROP5 (upper panel) and ROP18 (lower panel). Vertical lines indicate excision of irrelevant tracks from the images of the three gels.
(EPS)

Table S1 Primers used for site-directed mutagenesis of Irga6. All primers shown represent the top strand and are in 5′-3′-orientation.
(DOC)

Table S2 Native and experimental N-termini of IRG proteins in GST fusions. Shown are the sequences at the N-termini of IRG proteins expressed either as GST-fusion proteins in bacterial expression constructs or cleaved from the fusion as a near-native protein (Irga6). Residues in lower case are derived from the protease cleavage site and the polylinker. GST-Irga6 and GST-Irgb6 contain a thrombin cleavage site, and GST-Irgb10 contains a TEV-protease cleavage site. The protease cleavage sites are indicated by hyphens in the sequence. The first residues of the native protein are in capital letters. The N-terminal residues of the native open reading frames (ORF) are given below.
(DOC)

Table S3 Statistical data for Figure 3. The tables show the original data for the two independent experiments (I, black, Table S3A; II, grey, Table S3B) shown in Figure 3, counting vacuoles from strains RH, RHΔ*rop5*, and RHΔ*rop18* loaded with Irga6 or Irgb6 in IFNγ-induced C57BL/6 MEFs. The data for Irga6 and Irgb6 are plotted as percentages in Figure 3B and 3E, respectively. Probabilities that data for RHΔ*rop5* and RHΔ*rop18* are drawn from the same population as data from the parental strain RH were calculated by Fisher's exact test in 2×2 contingency tables, as shown in the last column.
(DOC)

Table S4 Statistical data for Figure 5. The tables show the original data for the two independent experiments (I, black, Table S4A; II, grey, Table S4B) shown in Figure 5, counting vacuoles from strains RH, RHΔ*rop5*, RHΔ*rop5*+A_III_/A_III_, and RHΔ*rop5*+A_III_/B_III_ loaded with Irga6 or Irgb6 in IFNγ-induced C57BL/6 MEFs. The data for Irga6 and Irgb6 are plotted as percentages in Figure 5B and 5D, respectively. Probabilities that data for RHΔ*rop5* and the two transgenic strains are drawn from the same population as data from the parental strain RH were calculated by Fisher's exact test in 2×2 contingency tables, as shown in the last column.
(DOC)

## Abbreviations

GST: glutathione S-transferase
IFNγ: interferon-γ
MEFs: mouse embryonic fibroblasts
MS: mass spectrometry
NMR: nuclear magnetic resonance
PVM: parasitophorous vacuole membrane
*T*. *gondii*: *Toxoplasma gondii*
