# Supplementary material for: A Toxoplasma gondii Pseudokinase Inhibits Host IRG Resistance Proteins
Source: PLoS Biol. 2012 Jul 10;10(7):e1001358. doi: 10.1371/journal.pbio.1001358 (PMC3393671; doi:10.1371/journal.pbio.1001358)
Supplement: Table S1 — Primers used for site-directed mutagenesis of Irga6. All primers shown represent the top strand and are in 5′-3′-orientation. (DOC) [file pbio.1001358.s004.doc]

**Table S1. Primer list for mutagenesis of Irga6**

| **Mutation** | **Primer 5`-3** |
| --- | --- |
| G195R | CAAATGAAGCAGATCGCAAACCTCAAACC |
| P197H | GCAGATGGCAAACATCAAACCTTTGACAAAG |
| Q198E | GCAGATGGCAAACCTGAAACCTTTGACAAAG |
| V205R | GACAAAGAAAAGCGCCTGCAGGACATCCGC |
| D208R | GAAAAGGTCCTGCAGCGCATCCGCCTTAAC |
| I209R | GGTCCTGCAGGACCGCCGCCTTAACTGTGTG |
| N212R | GGACATCCGCCTTCGCTGTGTGAACACC |
| C213R | GGACATCCGCCTTAACCGTGTGAACACCTTTAGGG |
| N220R | CCTTTAGGGAGCGTGGCATTGCTGAGCC |
