# Supplementary material for: A Toxoplasma gondii Pseudokinase Inhibits Host IRG Resistance Proteins
Source: PLoS Biol. 2012 Jul 10;10(7):e1001358. doi: 10.1371/journal.pbio.1001358 (PMC3393671; doi:10.1371/journal.pbio.1001358)
Supplement: Table S2 — Native and experimental N-termini of IRG proteins in GST fusions. Shown are the sequences at the N-termini of IRG proteins expressed either as GST-fusion proteins in bacterial expression constructs or cleaved from the fusion as a near-native protein (Irga6). Residues in lower case are derived from the protease cleavage site and the polylinker. GST-Irga6 and GST-Irgb6 contain a thrombin cleavage site, and GST-Irgb10 contains a TEV-protease cleavage site. The protease cleavage sites are indicated by hyphens in the sequence. The first residues of the native protein are in capital letters. The N-terminal residues of the native open reading frames (ORF) are given below. (DOC) [file pbio.1001358.s005.doc]

**Table S2. Native and experimental N-termini of IRG proteins.**

| **Protein** | **N-terminus** |
| --- | --- |
| Irga6 | GSPGIPGSTT-MGQL... |
| **GST**-Irga6 | **GST**-LVPRGSPGIPGSTT-MGQL... |
| **GST**-Irgb6 | **GST**-LVPRGSPGIPGSTT-MAWA... |
| **GST**-Irgb10 | **GST**-LENLYFQG-TGGQS... |
| **ORF** |  |
| *irga6* | MGQL... |
| *irgb6* | MAWA... |
| *irgb10* | MGQS... |
