# Supplementary material for: A Toxoplasma gondii Pseudokinase Inhibits Host IRG Resistance Proteins
Source: PLoS Biol. 2012 Jul 10;10(7):e1001358. doi: 10.1371/journal.pbio.1001358 (PMC3393671; doi:10.1371/journal.pbio.1001358)
Supplement: Table S3 — Statistical data for Figure 3. The tables show the original data for the two independent experiments (I, black, Table S3A; II, grey, Table S3B) shown in Figure 3, counting vacuoles from strains RH, RHΔrop5, and RHΔrop18 loaded with Irga6 or Irgb6 in IFNγ-induced C57BL/6 MEFs. The data for Irga6 and Irgb6 are plotted as percentages in Figure 3B and 3E, respectively. Probabilities that data for RHΔrop5 and RHΔrop18 are drawn from the same population as data from the parental strain RH were calculated by Fisher's exact test in 2×2 contingency tables, as shown in the last column. (DOC) [file pbio.1001358.s006.doc]

Supplementary Table 3

Supplementary Table S3A

| Expt I | Strain | Vacuoles | Vacuoles | Total | % + | Fisher’s Exact Test |
| --- | --- | --- | --- | --- | --- | --- |
|  |  | loaded | unloaded |  |  | p = |
| (1) Irga6 | RH | 51 | 53 | 104 | 49 |  |
| (2) Irga6 | RHΔ*rop5* | 69 | 29 | 98 | 69 | (1) vs (2) <0.01 |
| (3) Irga6 | RHΔ*rop18* | 90 | 15 | 105 | 14 | (1) vs (3) <0.001 |
|  |  |  |  |  |  |  |
| (4) Irgb6 | RH | 4 | 100 | 104 | 4 |  |
| (5) Irgb6 | RHΔ*rop5* | 71 | 27 | 98 | 71 | (4) vs (5) <0.001 |
| (6) Irgb6 | RHΔ*rop18* | 24 | 81 | 105 | 23 | (4) vs (6) <0.001 |

Supplementary Table S3B

| Expt II | Strain | Vacuoles | Vacuoles | Total | % + | Fisher’s Exact Test |
| --- | --- | --- | --- | --- | --- | --- |
| I |  | loaded | unloaded |  |  | p = |
| (1) Irga6 | RH | 59 | 42 | 101 | 59 |  |
| (2) Irga6 | RHΔ*rop5* | 76 | 24 | 100 | 76 | (1) vs (2) <0.01 |
| (3) Irga6 | RHΔ*rop18* | 92 | 8 | 100 | 92 | (1) vs (3) <0.001 |
|  |  |  |  |  |  |  |
| (4) Irgb6 | RH | 14 | 90 | 104 | 14 |  |
| (5) Irgb6 | RHΔ*rop5* | 78 | 22 | 100 | 78 | (4) vs (5) <0.001 |
| (6) Irgb6 | RHΔ*rop18* | 61 | 39 | 101 | 61 | (4) vs (6) <0.001 |

Legend to Supplementary Table 3A and 3B.

The tables show the original data for the two independent experiments (I, black; II, grey) shown in Fig. 3, counting vacuoles from strains RH, RHΔ*rop5* and RHΔ*rop18* loaded with Irga6 or Irgb6 in IFNγ-induced C57BL/6 MEFs. The data for Irga6 and Irgb6 are plotted as percentages in Figs 3B and 3E. Probabilities that data for RHΔ*rop5* and RHΔ*rop18* are drawn from the same population as data from the parental strain RH were calculated by Fisher’s Exact Test in 2x2 contingency tables, as shown in the last column.
