# Supplementary material for: A Toxoplasma gondii Pseudokinase Inhibits Host IRG Resistance Proteins
Source: PLoS Biol. 2012 Jul 10;10(7):e1001358. doi: 10.1371/journal.pbio.1001358 (PMC3393671; doi:10.1371/journal.pbio.1001358)
Supplement: Table S4 — Statistical data for Figure 5. The tables show the original data for the two independent experiments (I, black, Table S4A; II, grey, Table S4B) shown in Figure 5, counting vacuoles from strains RH, RHΔrop5, RHΔrop5+AIII/AIII, and RHΔrop5+AIII/BIII loaded with Irga6 or Irgb6 in IFNγ-induced C57BL/6 MEFs. The data for Irga6 and Irgb6 are plotted as percentages in Figure 5B and 5D, respectively. Probabilities that data for RHΔrop5 and the two transgenic strains are drawn from the same population as data from the parental strain RH were calculated by Fisher's exact test in 2×2 contingency tables, as shown in the last column. (DOC) [file pbio.1001358.s007.doc]

Table S4A

| Data Expt 1 | Strain | Vacuoles | | | % + | Fisher’s Exact Test |
| --- | --- | --- | --- | --- | --- | --- |
|  |  | + | - | Total |  | p = |
| (1) Irga6 | RH | 71 | 48 | 119 | 61 |  |
| (2) Irga6 | RHΔ*rop5* | 89 | 14 | 103 | 87 | (1) vs (2) p<0.001 |
| (3) Irga6 | RHΔ*rop5*  *+ROP5A+A* | 72 | 31 | 103 | 70 | (1) vs (3) NS |
| (4) Irga6 | RHΔ*rop5*  *+ROP5A+B* | 55 | 57 | 112 | 49 | (1) vs (4) NS |
|  |  |  |  |  |  |  |
| (5) Irgb6 | RH | 10 | 109 | 119 | 8 |  |
| (6) Irgb6 | RHΔ*rop5* | 87 | 16 | 103 | 85 | (5) vs (6) p<0.001 |
| (7) Irgb6 | RHΔ*rop5*  *+ROP5A+A* | 70 | 33 | 103 | 70 | (5) vs (7) p<0.001 |
| (8) Irgb6 | RHΔ*rop5*  *+ROP5A+B* | 8 | 104 | 112 | 7 | (5) vs (8) NS |

Table S4B

| Data Expt 2 | Strain | Vacuoles | | | % + | Fisher’s Exact Test |
| --- | --- | --- | --- | --- | --- | --- |
|  |  | + | - | Total |  | p = |
| (1) Irga6 | RH | 160 | 66 | 226 | 71 |  |
| (2) Irga6 | RHΔ*rop5* | 145 | 59 | 204 | 71 | (1) vs (2) NS |
| (3) Irga6 | RHΔ*rop5*  *+ROP5A+A* | 66 | 44 | 110 | 66 | (1) vs (3) NS |
| (4) Irga6 | RHΔ*rop5*  *+ROP5A+B* | 28 | 82 | 110 | 25 | (1) vs (4) p<0.001 |
|  |  |  |  |  |  |  |
| (5) Irgb6 | RH | 14 | 212 | 226 | 6 |  |
| (6) Irgb6 | RHΔ*rop5* | 133 | 71 | 204 | 65 | (5) vs (6) p<0.001 |
| (7) Irgb6 | RHΔ*rop5*  *+ROP5A+A* | 63 | 47 | 110 | 57 | (5) vs (7) p<0.001 |
| (8) Irgb6 | RHΔ*rop5*  *+ROP5A+B* | 4 | 106 | 110 | 4 | (5) vs (8) NS |

Legend to Table 4A and 3B.

The tables show the original data for two independent experiments counting vacuoles from strains RH, RHΔ*rop5*, RHΔ*rop5*+ROP5A+A and RHΔ*rop5*+ROP5A+B*,* loaded with Irga6 or Irgb6 in IFNγ-induced C57BL/6 MEFs. The combined data for Irga6 and Irgb6 are plotted as percentages in Figs 5B and 5D. Probabilities that data for RHΔ*rop5* and the RHΔ*rop5* transgenic strains are drawn from the same population as data from the parental strain RH were calculated by Fisher’s exact test in 2x2 contingency tables, as shown in the last column.
